# The roles of gut microbiota and its metabolites in diabetic nephropathy

**DOI:** 10.3389/fmicb.2023.1207132

**Published:** 2023-07-27

**Authors:** Hui Zhao, Cheng-E Yang, Tian Liu, Ming-Xia Zhang, Yan Niu, Ming Wang, Jun Yu

**Affiliations:** ^1^Clinical Experimental Center, Xi’an Engineering Technology Research Center for Cardiovascular Active Peptides, the Affiliated Xi’an International Medical Center Hospital, Northwest University, Xi’an, Shaanxi, China; ^2^Faculty of Life Science and Medicine, Northwest University, Xi’an, Shaanxi, China; ^3^Department of Cardiology, Xi'an International Medical Center Hospital, Xi’an, Shaanxi, China; ^4^College of Food Science and Engineering, Northwest University, Xi’an, Shaanxi, China

**Keywords:** diabetic nephropathy, gut microbiota, microbial metabolites, gut–kidney axis, microbiota-targeted therapies

## Abstract

Diabetic nephropathy (DN) is a severe microvascular complication of diabetes, which increases the risk of renal failure and causes a high global disease burden. Due to the lack of sustainable treatment, DN has become the primary cause of end-stage renal disease worldwide. Gut microbiota and its metabolites exert critical regulatory functions in maintaining host health and are associated with many pathogenesis of aging-related chronic diseases. Currently, the theory gut–kidney axis has opened a novel angle to understand the relationship between gut microbiota and multiple kidney diseases. In recent years, accumulating evidence has revealed that the gut microbiota and their metabolites play an essential role in the pathophysiologic processes of DN through the gut–kidney axis. In this review, we summarize the current investigations of gut microbiota and microbial metabolites involvement in the progression of DN, and further discuss the potential gut microbiota-targeted therapeutic approaches for DN.

## 1. Introduction

Diabetes is an age-related disease, which results in high morbidity and mortality worldwide with an estimated morbidity rate of more than 10.5% in 2021 (Sun H. et al., 2022). Nowadays, the prevalence of diabets is increasing dramatically with the aging of the population (Hamzé et al., 2022). Diabetic nephropathy (DN), kidney injury due to diabetes, is the most common complications of diabetes, occurring in approximately 40% of people with diabetes (Chen et al., 2023). The number of people with diabetes is expected to increase from 537 to783 million over the next 24 years causing a rise in global DN prevalence (Tuttle et al., 2022). To date, the therapeutic approaches mainly include the lifestyle intervention and medication to control blood pressure and hyperglycemia (Warren et al., 2019). In addition, it is reported that the proportion of end-stage renal disease (ESRD) caused by DN is nearly 38.8, 23 and 31.2% in America China and India, respectively (Hussain et al., 2019; Zhang et al., 2020; Johansen et al., 2021). Once DN evolves into ESRD, it requires treatment with renal replacement therapy, which leads to high mortality and huge socioeconomic burden (Rustiasari and Roelofs, 2022). Therefore, new alternative strategies to protect against DN are urgently needed.

Recently, the gut microbiota has become a hotspot as a potential participant in the onset and progression of DN. The gut microbiota is represented by trillions of microorganisms, including bacteria, viruses, fungi and archaea in the intestine of humans (Fernandes et al., 2022), which exerts many functions of digesting food (Naimi et al., 2021), regulating the immune system (Spindler et al., 2022), synthesizing vitamins (Zafar and Saier, 2021), removing pathogens (Silwal et al., 2021) and maintaining gut function (Sun R. Y. et al., 2022). The gut microbiota of healthy adults belongs to Firmicutes, Bacteroidetes, Proteobacteria and Actinobacteria, of which Firmicutes and Bacteroidetes are dominant with a proportion of approximately 90% (Eckburg et al., 2005). Under the normal physiological circumstances, the gut microbiota has a relatively stable species and numbers to protect the structure of intestinal mucosal barrier (Lei et al., 2022). However, the intestinal barrier can be damaged, once the homeostasis of gut microbiota is disrupted, which may potentially increase the risk of developing DN.

The gut microbial dysbiosis may cause the endotoxin and pathogen to cross the intestinal barrier, resulting in the inflammation and oxidative stress, and further accelerating kidney damage (Su et al., 2022). In addition, the gut microbial dysbiosis may lead to the alteration of microbial metabolites, which are considered as important substances to regulate life activity and metabolism, and participant in the onset and progression of various diseases (Jaye et al., 2022). In recent years, the application of high-throughput sequencing technology helps us to better understand the relationship between microbiome and its host (Jo et al., 2020). The gut microbiota and microbial metabolites may have pathogenic or beneficial effects on the host (Zheng et al., 2021). Herein, we reviewed the recent investigations and highlighted the alteration and important regulatory function of gut microbiota and their metabolites in the onset and progression of DN. Besides, novel strategies associated with the microbiota-targeted therapies, which will pave the way toward new renoprotective studies for DN, are further discussed.

## 2. The pathophysiology of DN

**Figure 1:**
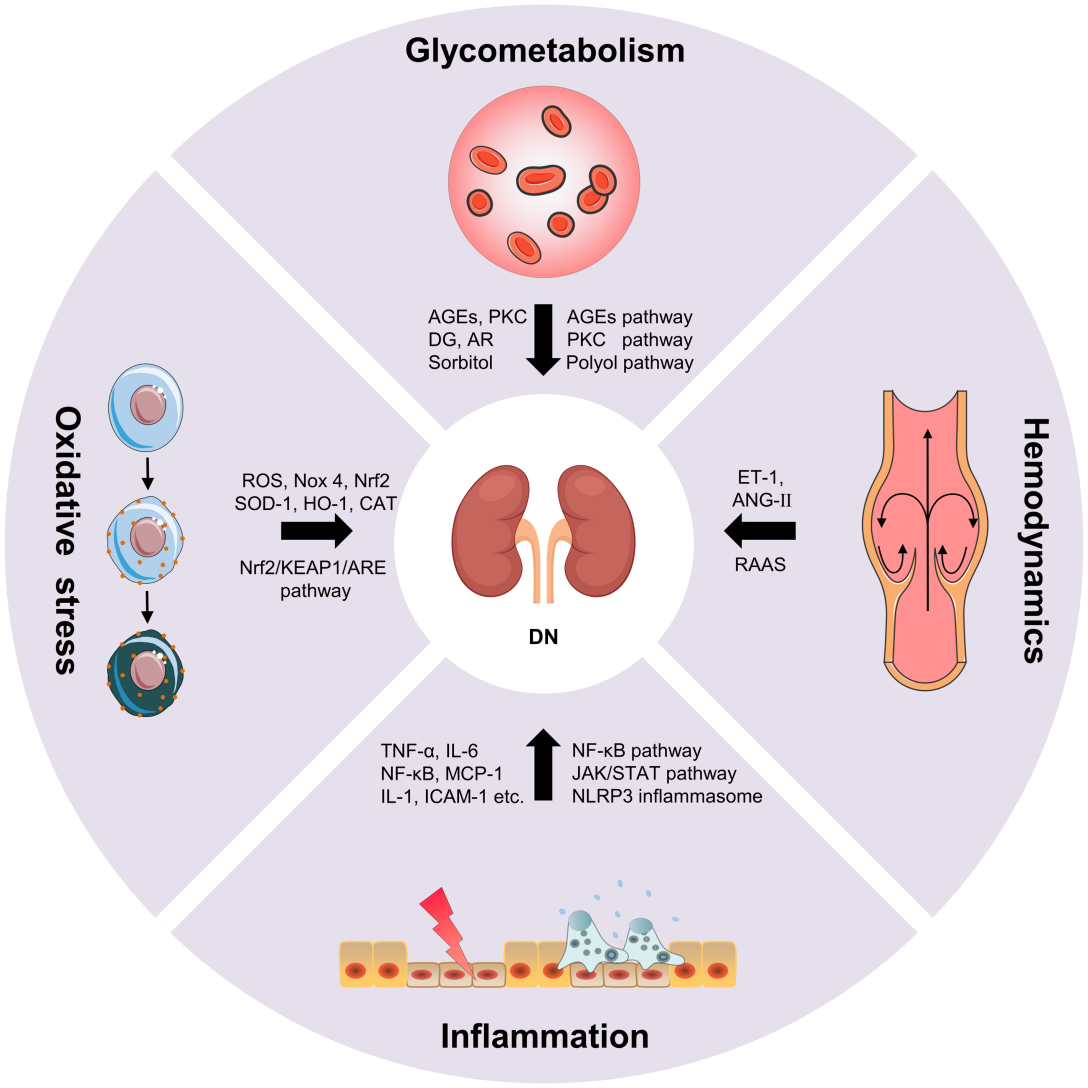
The pathogenesis of diabetic nephropathy (DN).

Diabetic nephropathy is characterized by glomerular basement membrane thickening, mesangial cell hypertrophy, podocyte loss, glomerulosclerosis and tubulointerstitial fibrosis, which eventually lead to progressive albuminuria and decline in estimated glomerular filtration rate (eGFR; Forst et al., 2022). The pathogenesis of DN is complex, including the disturbance of glycometabolism, abnormal hemodynamics, inflammation and oxidative stress. These mechanisms are interrelated, which together result in the progression of DN (Figure 1; Samsu, 2021).

### 2.1. Disturbance of glycometabolism

The disturbance of glycometabolism is the major characteristics of patients with DN, which mainly involves advanced glycation end products (AGEs) pathway, protein kinase C (PKC) pathway and polyol pathway (Jung and Yoo, 2022). AGEs are glycated products between reducing sugars and biomacromolecules (proteins, lipids, and nucleic acids) through a serious of non-enzymatic reactions (Ying et al., 2021). AGEs can directly damage the kidney, as well as cause the activation of various growth factors and cytokines by binding to the receptor of AGEs (RAGE). The persistent hyperglycemia in diabetic patients leads to the massive deposition of AGEs in endothelial cells, glomerular basement membrane and podocytes, directly damaging the normal structure of the glomerulus, impairing filtration function, and leading to the production of proteinuria (Wu et al., 2021). On the other hand, the accumulation of AGEs can bind to RAGE in kidney cells, which further leads to the intracellular inflammation and oxidative stress signaling pathways, such as nuclear factor-κB (NF-κB), phosphatidylinositide 3-kinase/protein kinase B (PI3K/Akt) and mitogen-activated protein kinase/extracellular signal-regulated kinase (MAPK/ERK), etc. In turn, oxidative stress and chronic inflammation promotes the generation of AGEs in kidney cells under hyperglycemic circumstances causing further aggravation of renal damage. The positive feedback regulation between AGEs/RAGE and downstream pathways results in a vicious cycle during the onset and progression of DN (Wu et al., 2021). Additionally, AGEs can directly activate the complement system and trigger pro-inflammatory, and causes mesangial cells, endothelial cells and podocytes to secrete inflammatory mediators such as cytokines, chemokines, and adhesion molecules. Whereafter, the monocytes and macrophages were activated and recruited, resulting in further inflammatory cascade response, ultimately leading to renal injury and tubulointerstitial fibrosis (Chen et al., 2022). PKC is abundant in human tissues, and participating in regulating cell proliferation, differentiation and apoptosis. The increase of intracellular blood glucose level leads to the increase of diacylglycerol (DG) synthesis and activation of PKC, thus inducing the production of the signal molecules such as endothelin-1 (ET-1), transforming growth factor-β1 (TGF-β1), interleukin-1 (IL-1) and reactive oxygen species (ROS) in kidney cells. Consequently, the increase in ET-1 results in the injury of endothelial cells. The overproduction of TGF-β1 leads to the accumulation of glomerular extracellular matrix and the hypertrophy of kidney cells. The overexpression of IL-1 and ROS causes the inflammation and oxidative stress in the kidney. These results collectively lead to the alteration in glomerular basement membrane structure, and increases of the glomerular capillary permeability and glomerular injury (Sheng et al., 2018). Additionally, the glucose can be converted into sorbitol by aldose reductase (AR) *via* polyol pathway under the diabetic circumstance. Sorbitol is prone to accumulate in kidney cells causing permeability alteration, cell swelling and rupture, which ultimately aggravate mesangial cells hypertrophy, glomerulosclerosis and tubulointerstitial fibrosis (Shen and Wang, 2021).

### 2.2. Hemodynamics

Abnormal hemodynamics is also an important cause of DN (Yang and Xu, 2022). The renin-angiotensin-aldosterone system (RAAS) has been shown to play an important role in the progression of kidney disease. The activation of RAAS leads to the changes of intrarenal hemodynamics, which further causes the structural changes of glomerulus and tubulointerstitium (Nishiyama and Kobori, 2018). Podocytes have been proved to produce many RAAS components and express its receptors. RAAS regulates systemic blood pressure through a homeostatic feedback loop under normal circumstances. As a key component of RAAS, angiotensin II (ANG-II) mainly plays a role in the kidney (Silva et al., 2021). The RAAS is over-activated, the ANG-II and aldosterone are increased under the hyperglycemic circumstance, which leads to the elevation of blood pressure and the destruction of sodium balance. Chronic glomerular hypertension, hyperperfusion and hyperfiltration result in glomerular sclerosis and renal interstitial fibrosis (Malek et al., 2021). Intrarenal Ang-II is a cytokine that contributes to kidney damage by multiple pathways, including increasing glomerular capillary permeability, stimulating mesangial cell hypertrophy, inducing ECM synthesis, as well as promoting macrophage and inflammatory cells infiltration (Samsu, 2021). Ang-II can also activate DG-PKC pathway, and induce ET-1 and ROS generation causing vasoconstriction and lipid peroxidation to deposit on glomerular intima. The disturbance of lipid metabolism results in the atherosclerosis and atheromatous plaque formation, which leads to the decrease of blood flow velocity and the increase of blood viscosity, thus causing glomerular hemodynamic abnormalities (Heilig et al., 2013; Roscioni et al., 2014). Additionally, as a potent vasoconstrictor of the efferent arteriole, the increase of ET-1 promotes the elevation of intrarenal blood pressure, which causes mesangial cells hypertrophy and ECM accumulation. Meanwhile, ET-1 can also increase the glomerular permeability by targeting its receptor, resulting in the aggravating albuminuria and DN progression (Ergul, 2011). The mechanical strain and shear stress, resulting from altered hemodynamics, lead to endothelial cell and epithelial cell injury, which accelerates the occurrence and development of proteinuria (Bai et al., 2022).

### 2.3. Inflammation

Hyperglycemia, metabolic disorder and abnormal hemodynamics can cause cellular injury and induce the inflammatory mediators, including tumor necrosis factor-α (TNF-α), interleukin-6 (IL-6), chemokines and adhesion molecules, etc. This leads to the activation and recruitment of the macrophages and monocytes to the kidney. The accumulation of macrophages produces ROS, pro-inflammatory factors, chemokines and metalloproteinases, which further aggravated kidney damage (DeFronzo et al., 2021; Jung and Yoo, 2022). DN inflammation is a complex integration of innate immune responses, in which macrophages are the main immune cells involved in DN renal injury, with two activation states: M1 and M2. Macrophages in M1 state release inflammatory factors and promote inflammation. Macrophages in M2 state are involved in anti-inflammation response and tissue repair. The activation level of M1 and M2 macrophages is closely associated with tissue microenvironment, disease status, etc. (Landis et al., 2018). Cytokines, such as TNF-α and IL-6, are essential pathogenic factor of DN. The mast cells can secrete TNF-α to induce renal injury when the inflammatory lesions occur in the renal. TNF-α can activate the apoptotic signaling pathway by targeting TNFR1 or activate downstream NF-κB to induce the production of other cytokines by targeting TNFR2 (Sun and Kanwar, 2015). Moreover, the combination of TNF-α and TNFR2 can also promote the production of ROS, which induced microvascular endothelial cell damage, reduced barrier function, and led to the production of proteinuria. IL-6 can act on interstitial cells near capillaries and release collagenase and other extracellular proteases, which leads to the degradation and leakage of glucoproteinases and the occurrence of microalbuminuria, thus promoting the occurrence and development of DN (Mansoor et al., 2022). Chemokines, such as monocyte chemoattractant protein 1 (MCP-1) can cause glomerular basement membrane injury and stimulate TGF-β1 to participate in glomerular sclerosis and tubulointerstitial fibrosis (Xie et al., 2022). Adhesion molecules, such as intracellular adhesion molecule 1 (ICAM-1) can aggregate inflammatory cells through kinase reaction and cause inflammatory response. In addition, inflammation and the pathophysiology of DN involve complex signaling pathway activation (Bui et al., 2020), such as NF-κB pathway, The janus kinase/signal transducers and activators of transcription (JAK–STAT) pathway and nod-like receptor (NLR) family pyrin domain containing 3 (NLRP3) inflammasome pathway. NF-κB, a transcription factor that involved in inflammatory response, can be activated by hyperglycemic conditions, leading to RAAS activation, AGEs accumulation and nicotinamide adenine dinucleotide phosphate (NADPH)-dependent oxidative stress. NF-κB activation is related to proteinuria, which can further stimulate NF-κB activation and form a vicious circle between NF-κB and proteinuria (Mezzano et al., 2004). The JAK–STAT is a critical pathway that responds to and mediates inflammatory molecules, which is significantly upregulated in glomerular cells of DN patients and is negatively associated with eGFR. Meanwhile, NF-κB can be activated by JAK–STAT signaling and further induced the transcription of proinflammatory factors, chemokines and adhesion molecules, etc. (Navarro-Gonzalez et al., 2011). NLRP3 inflammasome, a crucial regulator of chronic inflammatory response, is activated under the hyperglycemic circumstances. The activated NLRP3 inflammasome further mediates glomerular and tubular injury *via* releasing various inflammatory cytokines (Williams et al., 2022).

### 2.4. Oxidative stress

When the organism suffers from harmful stimulation, it will produce excessive ROS, which causes the imbalance of between the oxidation system and the antioxidant system and induce oxidative stress (Su et al., 2023). In DN, increase ROS production due to hyperglycemia can cause oxidative stress, which can directly damage podocytes, mesangial cells and endothelial cells, and lead to albuminuria and tubulo-interstitial fibrosis. Nicotinamide adenine phosphate dehydrogenase (NADPH) is an important source of ROS in diabetes. NADPH oxidase 4 (Nox 4) is the major isoform, which can be increased by hyperglycemia in the kidney (Pavlov et al., 2020). Hyperglycemia can also lead to glycosylation of antioxidant enzymes, which reduces enzyme activity and weakens the ability of the organism to clear free radicals. Oxidative stress interacts with almost every pathogenesis of DN. The combination of AGEs and RAGEs, PKC activation, polyol pathway and Ang-II can stimulate ROS production and induce oxidative stress (Tanase et al., 2022). Meanwhile, oxidative stress can cause kidney damage by activating other signal molecules such as Ang-II, PKC and TGF-β. In turn, the activation of these signaling molecules can cause oxidative stress and then induce renal injury (Samsu, 2021). Of note, Oxidative stress is closely associated with inflammatory response. In DN, ROS can activate NF-κB pathway to induce a large number of inflammatory mediators and trigger inflammatory response, which in turn aggravates oxidative stress damage (Winiarska et al., 2021). High glucose-induced oxidative stress can also cause macrophage infiltration that secretes pro-inflammatory factors and results in glomerular inflammation (Tuttle et al., 2022). Nuclear factor E2-related factor 2 (Nrf2) is an essential regulator of oxidative stress, which has attracted extensive attention in the antioxidant mechanism of DN in recent years. Nrf2 regulates the expression of antioxidant genes through Nrf2/KEAP1/ARE pathway, thus alleviating oxidative stress in DN (Tanase et al., 2022). The increased ROS inhibited the activation of Nrf2, and antioxidant enzymes, such as superoxide dismutase (SOD)-1, heme oxygenase (HO)-1 and catalase (CAT; Huang Q. H. et al., 2017). Studies have shown that knockout of Nrf2 gene in type 2 diabetic mice will aggravate the oxidative stress-induced renal injury (Liu Y. X. et al., 2022).

## 3. The roles of gut microbiota in DN

### 3.1. The gut–kidney axis

**Figure 2:**
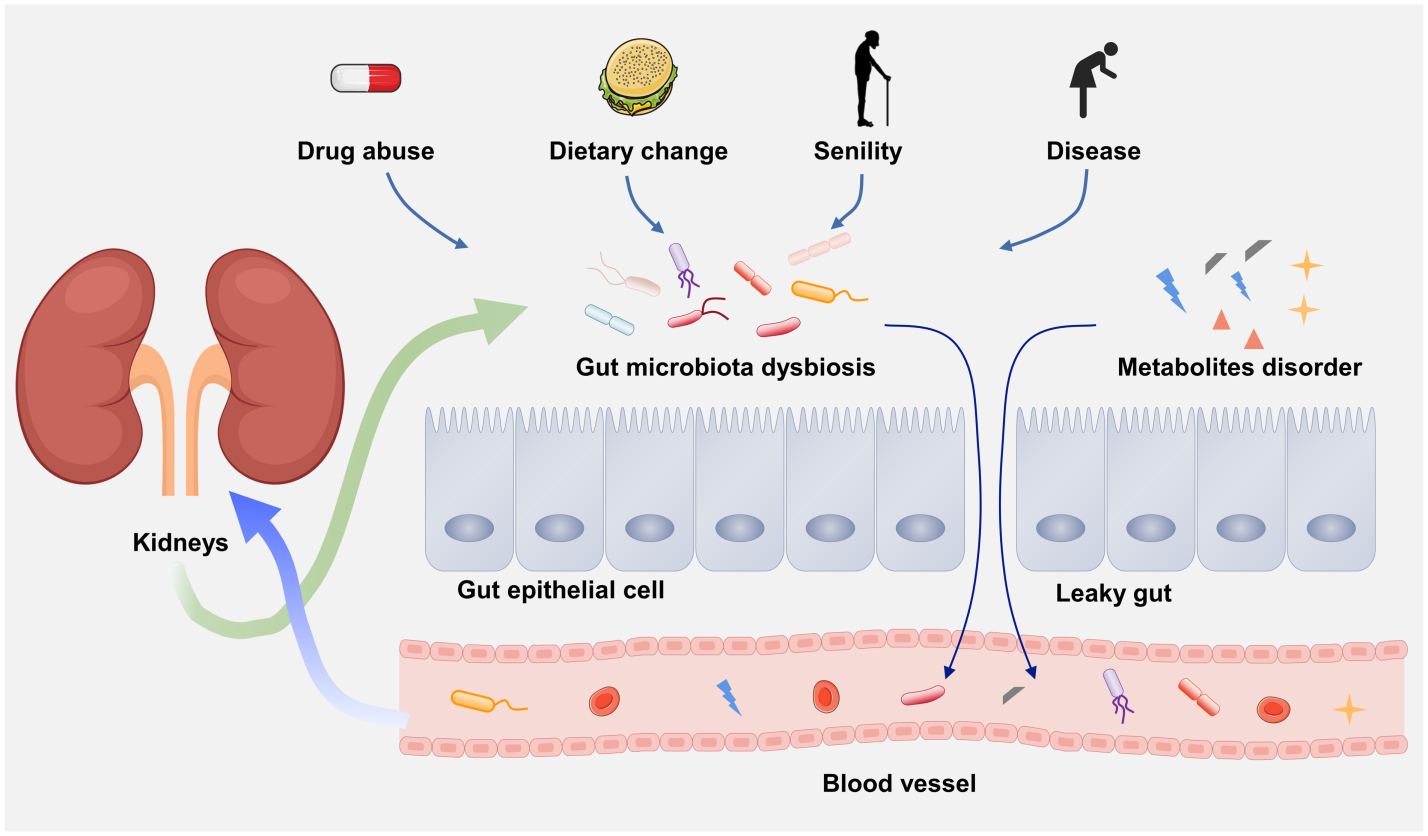
The gut–kidney axis.

In 2011, scientists first linked the kidney and gut, and clarify the influence of gut on chronic kidney disease (CKD), generally viewed as gut–kidney axis (Figure 2; Meijers and Evenepoel, 2011). Over the past decades, an increasing number of evidence has demonstrated that there is a bidirectional crosstalk between gut and kidney, namely, the pathophysiological alterations in the intestinal tract or the kidney can affect each other, resulting in lesions on the other side (Lobel et al., 2020). For example, due to the gut microbial dysbiosis, the translocation of intestinal microorganism may occur from gut to the kidney causing kidney damage (Stavropoulou et al., 2021). A latest study indicated that the renal injury of CKD rats can be effectively alleviated by regulating gut microbial dysbiosis and improving gut barrier (Hu et al., 2023). On the other hand, kidney diseases-associated gut microbial dysbiosis may lead to the impairment of gut barrier (Sun X. et al., 2022). Many nitrogen-containing organics released by impaired renal may across the gut barrier and promote the growth of intestinal pathogens. The metabolic wastes that cannot excrete by the kidney are prone to enter the intestunal lumen, thereby aggravating the gut microbial dysbiosis (Ni et al., 2022). Kidney diseases can also causes the alteration of gut microbiota composition. A systemic review have revealed that the α-diversity of the gut microbiota was significantly reduced and the β-diversity was more distinct in ESRD patients than healthy controls by recruiting 1,436 CKD patients and 918 healthy controls (Zhao et al., 2021). In addition, gut microbiota may also influence the progression of kidney diseases through microbial metabolites. For instance, (1) short-chain fatty acid (SCFAs) can promote immunosuppression and developing regulatory T cells (Tregs; Dang et al., 2021). Clinical investigations demonstrated that the Treg level in in peripheral blood of DN patients is lower than that of diabetic patients without kidney diseases (Wang M. et al., 2018). (2) Some uremic solutes, such as indoxyl sulfate (IS) and p-cresyl sulfate (PCS) may cause the deterioration of renal function through AHR activation (Hobby et al., 2019). (3) Trimethylamine-N-oxide (TMAO) is a well-known risk factor for CKD (Zixin et al., 2022). In short, kidney diseases and gut flora disorder can affect each other in a vicious circle. To clarify the underlying mechanism of this interconnection may contribute to understand disease etiologies and pathogenesis.

### 3.2. The disruption of intestinal barrier in DN

The intestinal tract is the largest tissue and organ that absorb water and nutrients. The intestinal barrier on the inner surface of the intestinal tract provides effective protection for the body against the invasion of harmful substances and pathogens in the external environment (Niewiem and Grzybowska-Chlebowczyk, 2022). The gut microbial dysbiosis in DN patients can cause increased intestinal permeability and damaged intestinal barrier, namely leaky gut (Linh et al., 2022). In particular, due to the decline of eGFR in DN patients, a large amount of ammonia generated by the hydrolysis of urea are reabsorbed by renal tubules, and subsequently enter the liver to resynthesize urea. The increased urea induces the decreased expression of tight junction proteins, including claudin-1, occludin and zonula occludens-1 (ZO-1), which results in the disruption of intestinal epithelial tight junction (Vaziri et al., 2013). The gut microorganism can enter the underlying tissue compartment due to the breakdown of intestinal epithelial tight junction, and further accelerating the intestinal barrier damage (Hu et al., 2022). Also, ammonia can change the diversity of gut microbiota, and destroy the composition and function by increasing intestinal pH (Khan et al., 2021).

Furthermore, toll like receptors (TLR) exert a critical role in the inflammatory process. Extensive evidence has demonstrated that TLR2/TLR4/NF-κB pathway participates in the inflammatory response by promoting pro-inflammatory cytokines (TNF-α, IL-1, IL-6, etc.) in DN (Wang et al., 2019). Lipopolysaccharide (LPS), surface antigen of gram-negative bacteria, leaks into portal circulation due to the breakdown of intestinal barrier, which further causes endotoxemia and inflammation *via* TLR2/TLR4/NF-κB signaling pathway (Zhang F. et al., 2021). Evidence has shown that TLR4-deficient mice with DN exhibit less NF-κB activation, albuminuria, renal dysfunction and interstitial macrophage infiltration (Lin et al., 2012). Therefore, the disruption of intestinal barrier leads to the binding of LPS to TLRs, thus activating NF-κB signaling pathway is the inflammatory mechanism of DN mediated by gut microbiota. In addition, other microbial metabolites, such as IS, PCS and cresol can also enter the blood due to the increase of intestinal permeability, causing renal damage and accelerating the development of DN (Lv et al., 2022).

### 3.3. Alterations of gut microbial composition in DN

Based on gut–kidney axis theory, the occurrence of kidney diseases usually results in gut microbial dysbiosis. Overall, the alterations of gut microbial composition in DN are characterized by an increased abundance of pernicious bacteria such as *Desulfovibrio* and *Coprobacillus*, and the decrease in probiotics such as *Lachnospira* and *Intestinibacter* (Table 1; Sheng et al., 2018; Su et al., 2022; Zhang L. L. et al., 2022). A recent study demonstrated that the intestinal microbiome richness and diversity of DN patients are far less than healthy subjects. Alterations in the abundance of gut microbiota, involving increases of *Megasphaera*, *Acidaminococcus* and *Lactobacillus* and decreases of *Tyzzerella_3*, *Roseburia* and *Lachnoclostridium* at the genus level, were observed in the fecal samples of DN patients (Du et al., 2021). Wang Y. W. et al. (2022) indicated that the enrichment of genus *Klebsiella*, *Citrobacter* and *Escherichia*, and the decrease of *Roseburia* may make the most contribution to gut microbial dysbiosis in DN by analyzing a large number of fecal samples of DN patients. Additionally, the discrepancies of the abundance and diversity of gut microbiota were also exist in different stages of DN (Du et al., 2021), suggesting that some characterized genera such as *Escherichia-Shigella*, *Megasphaera* and *Haemophilus* have potential to be novel microbial biomarkers for the diagnosis of DN. Tao et al. analyzed the gut microbiota composition of the fecal samples from DN patients, diabetic patients and healthy volunteers, and found that there are significant discrepancies among them. At the phylum to family level, Prevotellaceae is the bacteria with the highest proportion in healthy subjects, while Coriobacteriaceae is the most abundant bacteria in DN patients. The increased abundance of *Escherichia-Shigella* has been found in DN patients, whereas.

**Table 1:** Gut microbial alterations in diabetic nephropathy (DN).

Subjects	Increased	Decreased	Analytical methods	References
DN patients	Phylum level: Actinobacteria. Class level: Actinobacteria Bacilli, Coriobacteriia, Negativicutes. Order level: Betaproteobacteriales, Bifidobacteriales, Coriobacteriales, Lactobacillales, Selenomonadales. Family level: Atopobiaceae, Bifidobacteriaceae, Burkholderiaceae, Lactobacillaceae, Streptococcaceae, Tannerellaceae, Veillonellaceae. Genus level: *Acidaminococcus*, *Lactobacillus*, *Megasphaera*, *Mitsuokella*, *Olsenella*, *Prevotella_7*, *Sutterella*.	Class level: Alphaproteobacteria, Clostridia. Order level: Chitinophagales, Clostridiales, Rhizobiales, Xanthomonadales. Family level: Chitinophagaceae, Lachnospiraceae, Rhodanobacteraceae. Genus level: *Lachnoclostridium*, *Roseburia*, *Tyzzerella_3.*	16S rDNA	Du et al. (2021)
DN patients	Phylum level: Proteobacteria, Verrucomicrobia, Fusobacteria. Family level: Moraxellaceae, Xanthomonadaceae, Enterobacteriaceae, Pasteurellaceae, Alcaligenaceae, Verrucomicrobiaceae, Fusobacteriacea, Leptotrichiacene. Genus level: *Acinetobacter*, *Enhydrobacter*, *Stenotrophomonas*, *Citrobacter*, *Cronobacter*, *Enterobacter*, *Erwinia*, *Escherichia*, *Klebsiella*, *Pantoea*, *Proteus*, *Serratia*, *Trabulsiella*, *Actinobacillus*, *Achromobacter*, *Aggregatibacter*, *Sutterella*, *Akkermansia*, *Fusobacterium*, *Leptotrichia*.	/	16S rRNA	Salguero et al. (2019)
DN patients	Family level: Enterobacteriaceae.	/	16S rDNA qRT-PCR	Gradisteanu et al. (2019)
DN patients	Genus level: *Anaerococcus*, *Clostridium*, *Desulfitobacter*, *Enterococcus*, *Streptococcus*, *Desulfovibrio*, *Enterobacter*, *Escherichia*, *Klebsiella*, *Proteus*, *Pseudomonas*, *Acinetobacter* and *Citrobacter*.	Genus level: *Bifidobacterium.*	16S rRNA	Al-Obaide et al. (2017)
DN patients	Genus level: *Escherichia*–*Shigella*	Genus level: *Prevotella_9.*	16S rRNA	Tao et al. (2019)
DN patients	Species level: *Bacteroides stercoris*, *Prevotella* sp. *MSX73*, *Barnesiella*, *Alistipes ihumii*, *Bacteroides stercoris CAG_120*, *Tannerella* sp. *CAG_51*, *Parabacteroides sp. 20_3*.	Order level: Pseudomonadales. Family level: Moraxellaceae. Genus level: *Acinetobacter*, *Lachnospira*, *Romboutsia*, *Intestinibacter*, *Prevotellamassilia.* Species level: *Acinetobacter baumannii*, *Roseburia intestinalis*, *Romboutsia timonensis*, *Bacteroides plebeius CAG_211*, *Clostridium sp. CAG_768*, *Fusobacterium varium*, *Clostridium sp. 26_22*, *Clostridium sp. CAG_269*, *Clostridium sp. CAG_780*, *Eubacterium sp. AF22_9*, *Roseburia sp. AM23_20*, *Intestinibacter bartlettii*, *Ruminococcus bicirculans*, *Prevotellamassilia timonensis.*	Metagenomics	Zhang L. L. et al. (2022)
DN rats	Phylum level: Actinobacteriota. Class level: Bacilli, Actinobacteria. Genus level: *Lactobacillus*, *Bifidobacterium*, *Dubosiella*, *Coriobacteriaceae_UCG-002*, *Faecalibaculum*.	Class level: Clostridia. Genus level: *Turicibacter*, *Romboutsia*, *Ruminococcus*, *Lachnospiraceae_UCG-008.*	16S rRNA	Zhang B. et al. (2022)
DN rats	Phylum level: Proteobacteria, Deferribacteres.	Phylum level: Verrucomicrobia. Genus level: *Lactobacillus*, *Bacteroides*, *Akkermansia*.	16S rRNA	Yang J. et al. (2020)
DN rats	Genus level: *Blautia*, *Roseburia*, *Paraprevotella*.	Genus level: *Bacteroides.*	16S rDNA	Lu et al. (2020)
DN rats	Phylum level: Firmicutes, Actinobacteria, Proteobacteria, Tenericutes. Genus level:*Bifidobacterium*, *Clostridium*, *Ruminococcaceae_UCG-014*, *Ruminococcus_1*, *Turicibacter. norank_f_Ruminococcaceae*, *unclassified_f_Ruminococcaceae*.	Phylum level: Bacteroidetes, Verrucomicrobia. Genus level: *Ruminococcaceae*, *Bacteroides*, *Akkermansia*, *Fusicatenibacter*, *Blautia. norank_f_Muribaculaceae.*	16S rRNA	Zhang et al.
DN rats	Phylum level: Bacteroidetes.	Phylum level: Proteobacteria, Firmicutes.	16S rRNA	Zhao et al. (2019)
DN rats	Phylum level: Proteobacteria, Deferribacteres. Genus level: *Mucispirllum*, *Prevotella*, *Lactobacillus.*	Phylum level: Actinobacteria, Bacteroidetes. Genus level: *Bifidobacterium*, *Paraprevotella.*	16S rRNA	Feng et al. (2019)
DN rats	Phylum level: Actinobacteria. Genus level: *Turicibacter*, *Blautia*, *Dorea*, *Coprobacillus.*	Phylum level: Firmicutes, Bacteroidetes, Proteobacteria. Genus level: *Adlercreutzia*, *Odoribacter*, *Prevotella*, *S24-7*, *Clostridium*, *Ruminococcus*, *Coprococcus*, *Roseburia*, *Oscillospira*, *Ruminococcus*, *Phascolarctobacterium*, *Sutterella*, *Acinetobacter*, *Pseudomonas*, *RF39.*	16S rRNA	Sheng et al. (2018)
DN rats	Genus level: *Candidatus_Saccharimonas*, *Treponema*, *Desulfovibrio.*	Genus level: *Lactobacillus*, *Ruminococcaceae UCG-005*, *Anaerovibrio*, *Bacteroides*, *Christensenellaceae_R-7_group.*	16S rRNA	Su et al. (2022)
DN rats	Genus level: *Lactobacillus*, *Phascolarctobacterium.*	/	16S rDNA	Hu et al. (2020)
DN mice	Genus level: *Oscillibacter.*	Genus level: *Bateroid*, *Odoribacter.*	16S rRNA	Deng et al. (2022)
DN mice	/	Genus level: *Bacteroides*, *Alistipes*, *Rikenella*, *Odoribacter*, *Parabacteroides*, *Alloprevotella.*	16S rRNA	Cai et al. (2020)
DN mice	Genus level: *Bacteroides*, *Alloprevotella*, *Enterococcus*, *Klebsiella*, *Kurthia*, *Rikenella*, *Paraprevotella.*	Genus level: *Lactobacillus.*	16S rRNA	Chen Q. et al. (2021)
DN mice	Genus level: *Bacteroides*, *Eubacterium*, *Roseburia.*	Phylum level: Proteobacteria, Verrucomicrobiota, Epsilonbacteraeota. Genus level: *Desulfovibrio.*	16S rRNA	Li et al. (2021)
DN mice	Genus level: *Turicibacter*, *Syntrophococcus.*	Phylum level: Saccharibacteria. Genus level: *Akkermansia*, *Mucispirillum*, *Candidatus Saccharimonas and Prevotella 2.*	16S rRNA	Luo L. M. et al. (2022)
DN mice	Genus level: *Negativibacillus*, *Rikenella.*	Genus level: *Akkermansia*, *Candidatus*, *Erysipelatoclostridium*, *Ileibacterium.*	16 s rDNA and metagenomics	Wu C. et al. (2022)

*Prevotella_9* is reduced at the genus level. These two genera can accurately distinguish DN patients from diabetic subjects (Tao et al., 2019). In brief, these clinical investigations demonstrated that gut microbiota may serve as predictor for the onset and progression of DN. However, these alterations to the microbial abundance and composition at the phylum, family and genus levels may not sufficient to adequately clarify the changes to the gut microbiota in DN patients, further investigations at the levels of species or strains are necessary in the future.

The Firmicutes/Bacteroidetes (F/B) ratio is relatively stable in healthy mammals, and the elevation or decline of the ratio frequently indicates the disease state. It is reported that the increased F/B ratio is associated with diabetes and obesity (Cai et al., 2020). Several studies indicated that F/B radio is significantly increased in the fecal sample of DN mice or rats (Feng et al., 2019; Yang J. et al., 2020; Su et al., 2022; Zhang Z. P. et al., 2022), while Zhao et al. (2019) reported the opposite trend. These findings suggested that it is not accurate to judge the disease state based simply on the F/B ratio. Moreover, the opposite changes of several genera have also been reported in different studies. For example, Lu et al. reported the increased abundance of *Roseburia* in DN rats, whereas Sheng et al. reported the decreased trend (Sheng et al., 2018; Lu et al., 2020). These opposite results may partly attributed to colonization at birth, age, gender, diet, and other factors (Gilbert et al., 2018). In particular, the discrepancy in gut microbiota still existed between DN mice with severe proteinuria (SP) and mild proteinuria (MP). The abundance of *Allobaculum* and *Anaerosporobacter* in the SP mice was higher than that in the MP mice, while the *Blautia* was more abundant in MP mice (Li Y. et al., 2020), suggesting that the alteration of gut microorganisms may associated with the DN progression. *Blautia* may has a beneficial anti-DN role (Hsu et al., 2022), while *Allobaculum* and *Anaerosporobacter* may accelerate the deterioration of renal function in DN. Despite the fact that the data from experimental animals cannot completely represent the disease state in humans, these results in animal models provide substantial evidence and supplement for the investigations of gut microbiota in DN patients.

### 3.4. Correlations between gut microbiota and DN phenotypes

Nowadays, an increasing number of studies have revealed the correlations between gut microbial alterations and DN phenotypes, including renal function, glucose metabolism, lipid metabolism, pathological changes of kidney, inflammatory cytokines and oxidative stress factors. These phenotypes are usually associated with disease progression, suggesting the potential role of gut microbiota in the diagnosis and therapy of DN. In terms of renal function, extensive studies have revealed the correlation between eGFR, serum creatinine (SCR), urinary albumin-to-creatinine ratio (UACR), 24-h urine protein (24-h UP) and gut microbiota in DN patients. For example, *Abiotrophia*, *G_norank_f_Peptococcaceae* (Zhang Q. et al., 2021) and *Ruminococcus torques group* (Chen W. H. et al., 2021) were negatively associated with eGFR in DN patients, while *Lachnospiraceae_NC2004_group* (Zhang Q. et al., 2021), Verrucomicrobia and *Subdoligranulum* (Tao et al., 2019) showed a positive correlation. Two specific taxa, including *Abiotrophia* and *G_norank_f_Peptococcaceae* (Zhang Q. et al., 2021) were reported to be positively correlated with SCR, while *Clostridium* sp. *26_22* (Zhang L. L. et al., 2022), *Lachnospiraceae_NC2004_group* (Zhang Q. et al., 2021) and *Parabacteroides* (Tao et al., 2019) exhibited a reverse correlation. Additionally, the abundance of *Citrobacter farmeri*, *Syntrophaceticus schinkii* (He et al., 2022), Bacteroidetes and Elusimicrobia (Zhao et al., 2020) are increased in DN patients with higher UACR, whereas the abundance of Firmicutes, Bacteroidetes, *Bacteroides* (Tao et al., 2019) and Actinobacteria (Zhao et al., 2020) are decreased. Negative correlation was also observed between *Blautia* and 24-h UP (Li Y. et al., 2020) in DN patients, whereas *Anaerosporobacter* (Li Y., et al., 2020), *Abiotrophia* (Zhang Q. et al., 2021), *Alistipes* and *Subdoligranulum* (Chen W. H. et al., 2021) showed a positive association. These findings suggested that gut microbiota may act as potential biomarker for the detection of renal function in DN patients. Identifying the specific microbiome may shed new light on the diagnosis of DN progress. However, the regulatory mechanisms of the gut microbiome on renal function are not well understood, which limits the further utilize of gut microbiota in DN. In the field of glucose metabolism, glycosylated hemoglobin (HbA1c) and fasting blood glucose (FBG) are often used to evaluate the correlation between intestinal flora and DN. Current investigations revealed that Firmicutes, *Faecalibacterium*, *Lachnoclostridium*, *Roseburia* (Tao et al., 2019) *g_norank_f_norank_o_Oscillospirales* and *g_unclassified_f_Ruminococcaceae* (Zhang Q. et al., 2021) were negatively correlated with HbA1c in DN patients. *Allobaculum* (Li Y. et al., 2020) was positively associated with FBG, while Firmicutes, Fusobacteria and *Fusobacterium* (Tao et al., 2019) showed a negative association with FBG, which means that the increase in *Allobaculum* abundance or the decrease in Firmicutes, Fusobacteria and *Fusobacterium* abundance may lead to the elevated glucose utilization and absorption in DN. Gut microbiota may play a role in the blood glucose control of DN patients as an auxiliary strategy in the future. *Escherichia-Shigella* was positively associated with body mass index (BMI; Tao et al., 2019). In terms of blood lipids, *Bacteroides*, *Lachnoclostridium* and *Lachnospiraceae bacterium 3 1 46FAA* were positively associated with total cholesterol (TC) in DN patients, whereas *L. mucosae* was reported to be negatively correlated with low density lipoprotein-cholesterol (LDL-C). Chen et al. demonstrated that *Lachnoclostridium*, *Bacteroides* and *Parabacteroides* were positively associated with triglyceride (TG) by analyzing the fecal sample of DN patients (Chen W. H. et al., 2021; He et al., 2022; Zhang L. L. et al., 2022). Since TC and TG are independent risk factors of in DN (Jansson Sigfrids et al., 2021; Liu L. et al., 2021), low level of TC and TG contribute to the favorable prognosis in DN patients. Nonetheless, there is currently no evidence to prove whether the associations are causal or not. As for renal pathological injury, DN rats with more severe renal tubular injury possess higher abundance of Bacteroidetes and Elusimicrobia, and lower abundance of Firmicutes and Actinobacteria. Elusimicrobia was reported to be positively associated with glomerularsclerosis (Zhao et al., 2020). This is the only study to indicate the relationship between renal pathological injury of DN patients and gut microbiota, and more investigations are needed to clarify their associations. Intriguingly, certain genera of gut microbiota were associated with inflammatory cytokines and oxidative stress factors in renal tissues. For example, *Bacteroides* was correlated with increased expression of TNF-α in DN mice (Chen Q. et al., 2021), and *Allobaculum* was associated with increased expression of superoxide dismutase (SOD) in DN rats (Su et al., 2022). Further correlations between gut microbiota and DN phenotypes are detailed in Table 2.

**Table 2:** Correlations between gut microbiota and DN phenotypes.

DN phenotypes	Positive correlation	Negative correlation	References
eGFR	Phylum level: Verrucomicrobia. Genus level: *Lachnospiraceae_NC2004_group*, *Subdoligranulum*	Genus level: *Abiotrophia*, *Ruminococcus torques group. G_norank_f_Peptococcaceae*	Tao et al. (2019), Chen W. H. et al. (2021), Zhang Q. et al. (2021)
SCR	Genus level: *Abiotrophia. G_norank_f_Peptococcaceae*	Genus level: *Lachnospiraceae_NC2004_group*, *Parabacteroides.* Species level: *Clostridium sp. 26_22*	Tao et al. (2019), Zhang L. L. et al. (2022b), Zhang Q. et al. (2021)
UACR	Phylum level: Bacteroidetes, Elusimicrobia. Species level: *Citrobacter farmer*, *Syntrophaceticus schinkii*	Phylum level: Firmicutes, Bacteroidetes, Actinobacteria. Genus level: *Bacteroides*	Tao et al. (2019), Zhao et al. (2020), He et al. (2022)
24-h UP	Genus level: *Anaerosporobacter*, *Abiotrophia*, *Alistipes*, *Subdoligranulum*	Genus level: *Blautia*	Li Y. et al. (2020), Chen W. H. et al. (2021), Zhang Q. et al. (2021)
HbA1c	/	Phylum level: Firmicutes. Genus level: *Faecalibacterium*, *Lachnoclostridium*, *Roseburia. G_norank_f_norank_o_Oscillospirales*, *g_unclassified_f_Ruminococcaceae*	Tao et al. (2019), Zhang Q. et al. (2021)
FBG	Genus level: *Allobaculum*	Phylum level: Firmicutes, Fusobacteria. Genus level: *Fusobacterium*	Tao et al. (2019), Li Y. et al. (2020)
BMI	Genus level: *Escherichia*–*Shigella*	/	Tao et al. (2019)
Blood lipids	Genus level: *Bacteroides* (TC), *Lachnoclostridium* (TC), *Lachnoclostridium* (TG), *Bacteroides* (TG), *Parabacteroides* (TG). Species level: *Lachnospiraceae bacterium 3 1 46FAA* (TC)	Species level: *L. mucosae* (LDL-C)	Chen W. H. et al. (2021), He et al. (2022), Zhang L. L. et al. (2022)
Tubular injury index	Phylum level: Bacteroidetes, Elusimicrobia	Phylum level: Firmicutes, Actinobacteria	Zhao et al. (2020)
Glomerularsclerosis	Phylum level: Elusimicrobia	/	Zhao et al. (2020)
Inflammatory cytokines	Genus level: *Treponema* (IL-6), *Bacteroides* (IL-6, NF-κB, TNF-α, IL-1β), *Lactobacillus* (IL-10)	Phylum level: Elusimicrobia (TNF-α). Genus level: *Candidatus_Saccharimona* (TNF-α), *Ruminococcaceae UCG-005* (IL-1β), *Christensenellaceae_R-7_group* (IL-1β), *Clostridiales_unclassified* (IL-10), *Megasphaera* (IL-6)	Chen Q. et al. (2021), Chen W. H. et al. (2021), Su et al. (2022), Zhang M. et al. (2022), Zhao et al. (2020)
Oxidative stress factors	Genus level: *Candidatus_Saccharimonas* (MDA, GSH-Px), *Ruminococcaceae UCG-005* (SOD), *Allobaculum* (SOD), *Bacteroides* (MDA), *Christensenellaceae_R-7_group* (SOD)	Genus level: *Lactobacillus* (MDA), *Anaerovibrio* (MDA), *Bacteroides* (SOD)	Su et al. (2022)

Overall, these studies suggested that the alterations of gut microbial composition and abundance may be associated with DN symptoms or pathological changes. Nevertheless, the causalities between gut microbiota and DN are still confusing, as these cross-sectional studies only focused on one point in time. The longitudinal studies are scarce. Therefore, longitudinal and prospective investigations are imminently required to clarify the cause-effect associations between gut microbiota and DN. In addition, some current studies were performed on animal models and the clinical investigations are relatively lacking. These studies may not correlate well with clinical results and need to be further confirmed in DN patients. Some clinical studies that reveal the correlations between intestinal flora and DN phenotypes only include a few subjects. For example, He et al. indicated the associations between the UACR level and intestinal microbiome by analyzing the fecal sample from 10 DN patients (He et al., 2022). This sample size seems insufficient to identify the correlations. A larger-scale clinical cohort might be warranted to recognize these correlations.

## 4. The roles of gut microbial metabolites in DN

**Figure 3:**
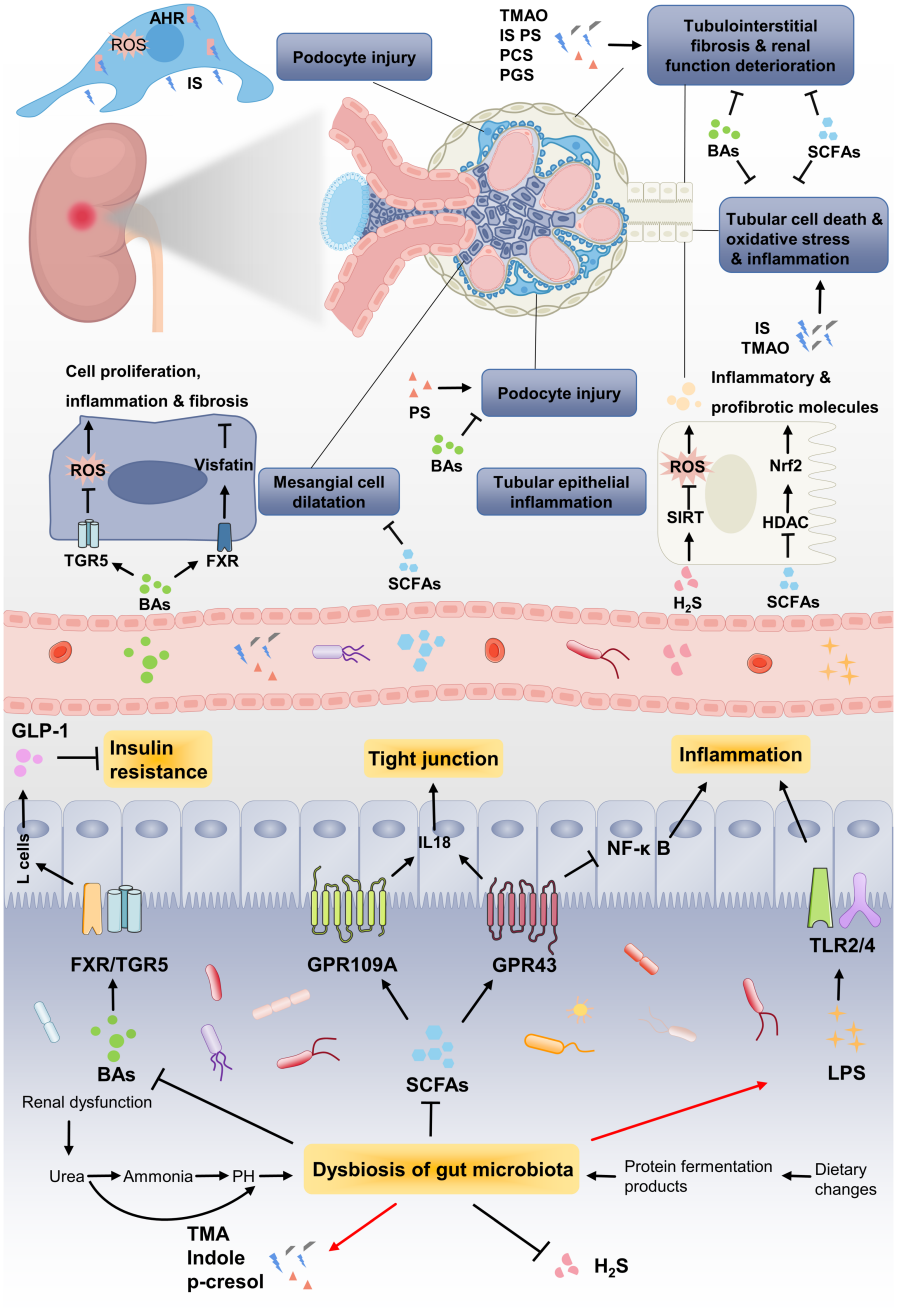
The pathogenic association between gut microbial metabolites and development of DN. Renal dysfunction and dietary changed lead to dysbiosis of gut microbiota, which further results in metabolic disorders of gut microbiota. Due to the decrease in SCFA-producing bacteria, the renoprotective effect of SCFAs through GPRs or HDAC is weakened, such as inflammation, tubulointerstitial fibrosis and glomerular injury. Gut microbiota dysbiosis lead to the reduction of BAs, which improving DN by alleviating mesangial cell dilatation and insulin resistance *via* targeting its receptors FXR/TGR5. Uremic toxins, including TMAO, IS, phenyl sulfate (PS), PCS and PGS, induces podocytes injury *via* binding to AHR or accelerates renal function deterioration. The enrichment of LPS caused by gut microbiota dysbiosis induces persistent inflammation by binding to TLRs, which steadily deteriorate DN.

Changes in gut microbiota can lead to the metabolic alterations in patients with DN. Multiple interactions between gut microbiota and human body are mediated through metabolites generated by intestinal microorganism. Current investigations have focused on revealing the relationship between DN and gut microbial metabolites including SCFAs, bile acids (BAs), TMAO, uremic toxins (UTs) and hydrogen sulfide (H_2_S). These metabolites affect the barrier function of intestinal epithelium by regulating the expression of receptors or activating transcription factors, thereby exerting critical functions in the onset and progression of DN (Figure 3).

### 4.1. Short-chain fatty acids

Short-chain fatty acids (SCFAs) are a group of fatty acids with no more than six carbon atoms derived from the microbial fermentation of indigestible dietary fiber in the colon and cecum, mainly including acetate, propionate and butyrate (approximately 90–95%; Lu P. C. et al., 2021; van der Hee and Wells, 2021). Being an essential signal molecules of human body, SCFAs are also involved in the regulation of metabolism by gut microbiota, such as cholesterol biosynthesis, glucose homeostasis and insulin sensitivity (Lee et al., 2020). Therefore, the decreased SCFAs are associated with the pathogenesis of multiple diseases, such as hypertension, hyperlipidemia and type 2 diabetes (Lu et al., 2022).

The perturbations of gut microbiota can lead to the decrease of SCFAs, resulting in the decreased insulin sensitivity and eventually the occurrence of type 2 diabetes (Makki et al., 2023). Recent studies have found that the fecal level of total SCFAs, acetate, propionate, butyrate and isovalerate are significantly decreased in DN patients. Serum and fecal SCFAs levels may be negatively correlated with renal function (Zhong et al., 2021; Li Y. et al., 2022). Mechanistically, gut microbiota-derived SCFAs exert their functions by activating trans-membrane G-protein-coupled receptors (GPCRs) of GPR41 and GPR43 or inhibiting histone acetylation (HDAC) to directly enter host cells (Moniri and Farah, 2021). SCFAs (acetate and butyrate) or GPR43 agonist can retard the HG-and LPS-induced glomerular mesangial cells (GMCs) proliferation, and then inhibit oxidative stress and inflammation. Exogenous SCFAs (especially butyrate) significantly ameliorated hyperglycemia and insulin resistance, improved renal function and inhibited renal fibrosis in high-fat diet (HFD) and streptozotocin (STZ)-induced DN mice *via* inhibiting GPR43-mediated NF-κ B signaling and oxidative stress (Huang W. et al., 2017; Huang et al., 2020). Meanwhile, Li Y. J. et al. (2020) demonstrated that dietary fiber can effectively ameliorate DN by increasing SCFAs-mediated activation of GPR43 and GPR109A. Sodium butyrate (NaB), an inhibitor of HDAC activity, is the most widely studied SCFA salt against DN (Gu et al., 2019). NaB supplementation can restore the composition of gut microbiota and enhance gut epithelial barrier integrity in diabetic mice (Xu et al., 2018). In addition, exogenous NaB administration ameliorated renal function, glucose and lipid metabolic disturbance, inflammation, fibrosis and oxidative injury by activating Nrf2 *via* inhibiting the activity of HDAC in DN mice (Dong et al., 2017; Du et al., 2020; Zhou et al., 2022). These findings suggested that SCFAs can improve DN by inhibiting oxidative stress, inflammation and renal fibrosis. SCFAs, especially butyrate, may serve as promising targets for the treatment of DN.

However, although SCFAs mostly exhibit a protective role in the progression of DN, some SCFAs can also bring about renal damage, if not properly regulated. For example, Hu et al. indicated that acetate can induce the disorder of cholesterol homeostasis by activating GPR43, thereby causing tubulointerstitial injury in DN (Hu et al., 2020). Moreover, it has been reported that excessive plasma acetate may result in renal injury by activating intrarenal renin-angiotensin system (RAS) in early DN rats (Lu et al., 2020). Therefore, the potential impacts of SCFAs in DN still need further study. The clinical and animal experiments of SCFAs in kidney diseases are still preliminary, and their underlying mechanisms in kidney diseases need to be confirmed by more studies.

### 4.2. Bile acids

Bile acids (BAs), major component of bile, are synthesized from cholesterol in the liver and transported to intestine (Perino et al., 2021). The primary BAs are then metabolized and transformed by the gut microbiota through a series of reactions, including deconjugation, dehydroxylation, oxidation and epimerization, to generate secondary BAs (Cai et al., 2022; Luo W. et al., 2022). Among them, Firmicutes, *Bacteroides*, *Lactobacillus*, *Bifidobacterium* and *Clostridium* play essential roles in the generation of secondary BAs (Lee et al., 2022). Most of these secondary BAs reenter the liver through the enterohepatic circulation, and the rest are excreted into feces (Bertolini et al., 2022). As critical signaling molecules, BAs have been confirmed to perform multiple biological functions, such as controlling energy (Wang S. P. et al., 2022), glucose (Makki et al., 2023) and lipid metabolism (Xu et al., 2022) and regulating immune system (Fuchs et al., 2022).

BAs significantly affect host metabolism by activating BA receptors, including the farnesoid X receptor (FXR) and the takeda G protein-coupled receptor 5 (TGR5; Thibaut and Bindels, 2022). Both FXR and TGR5 are highly expressed in the normal kidney tissues, but downregulated in DN, and the degree of decrease is associated with the inflammation and fibrosis (Wang X. X. X. et al., 2018). The renoprotective effect of FXR is mainly mediated by regulating lipid metabolism, oxidative stress, pro-inflammatory cytokines, and pro-fibrotic factors, thus inhibiting the increased proteinuria, podocyte loss, mesangial expansion and renal lipid accumulation (Masaoutis and Theocharis, 2019). In STZ-induced diabetic mice, knockout of FXR exacerbated diabetic kidney injury, increased the level of plasma lipid and accelerated fibrosis compared with diabetic wild-type mice (Wang et al., 2010). Oppositely, FXR agonist GW4064 inhibited the inflammation, fibrosis and cell proliferation by downregulating visfatin in HG-induced HMCs. Moreover, GW4064 reduced the level of blood glucose, albuminuria, BUN and Scr, attenuated glomerular injury and fibrosis in db/db mice, suggesting that FXR activation suppressed the progression of DN (Zhou et al., 2016).

TGR5, a membrane receptor activated by BAs, can directly regulate podocyte function. Evidence has shown that the renoprotective mechanisms of TGR5 activation mainly include inducing mitochondrial biogenesis, inhibiting oxidative stress and preventing renal lipid accumulation (Wang et al., 2016). Moreover, TGR5 activation induced the release of ileac glucagon-like peptide-1 (GLP-1; Ding et al., 2021), suggesting the potential therapeutic prospect of BAs against DN. In diabetic db/db mice, treatment with TGR5 agonist INT-777 significantly decreased proteinuria, glomerular mesangial expansion, glomerular podocyte injury and the accumulation of extracellular matrix proteins and macrophage (Wang et al., 2016). Consistently, Xiao et al. (2020) revealed that TGR5 activation alleviated pathological progression of DN by repressing inflammation through NF-κ B pathway. *In vitro* studies have also showed that the activation of TGR5 profoundly suppressed the expression of TGF-β1 and fibronectin (FN) in HG-induced GMCs, which can both promote renal fibrosis (Xiong et al., 2016; Yang et al., 2016).

Taken together, the activation of BA receptors showed a promising prospect in the prevention and therapy of diabetic kidney injury. FXR and TGR5 may play a synergistic effect in ameliorate kidney injury, inflammation and fibrosis. Therefore, FXR/TGR5 dual agonist may exhibit additional effects in the treatment of DN (Wang X. X. X. et al., 2018). BA receptors are expected to be therapeutic targets for the treatment of DN. BAs analogues may be a novel direction for the development of new drugs against DN.

### 4.3. Trimethylamine-N-oxide

Trimethylamine-N-oxide (TMAO) is mainly derived from dietary choline, L-carnitine and phosphatidylcholine, transforming these dietary nutrients to trimethylamine (TMA) through intestinal bacteria and further metabolized into TMAO by hepatic flavin monooxygenase 3 (FMO3; Yoo et al., 2021). *In vivo* studies have identified several microbiota associated with TMA/TMAO generation, including *Clostridium hathewayi*, *Providencia alcalifaciens*, *Escherichia fergusonii* and *Providencia rustigiani* (Romano et al., 2015). A large body of evidence has demonstrated that TMAO has served as a novel risk factor for diverse diseases, such as cardiovascular disease (Zhu et al., 2016), kidney disease (Chang et al., 2021) and type 2 diabetes mellitus (Li S.-Y. et al., 2022). Recently, the increased level of plasma TMAO has been reported to contribute to renal dysfunction and TMAO is reversely correlated with eGFR in the patients with type 2 diabetes. The gut microbiota of patients with type 2 diabetes is perturbed and the plasma TMAO level is elevated, which lead to the decrease of eGFR and the deterioration of renal function. The decreased eGFR causes further elevation of plasma TMAO (Adachi et al., 2019; Yu et al., 2022), suggesting that TMAO may serve as a biomarker associated with the occurrence of kidney diseases. Fang et al. indicated that the plasma level of TMAO is significantly higher in DN rats. Compared with DN rats on a normal diet, treatment with TMAO significantly exacerbated the renal function, and accelerated fibrosis and inflammation in DN rats (Fang et al., 2021). However, the addition of iodomethylcholine, a gut microbial choline TMA-lyase mechanism-based inhibitor, significantly reduced the plasma level of TMAO, ameliorated renal function and inhibited renal tubulointerstitial fibrosis in isoproterenol-induced CKD rats. Of note, iodomethylcholine also improved the choline diet-induced abnormal changed of gut microbial community, including *Lactobacillus*, *Bacteroides* and *Lachnospiraceae_UCG-002* (Gupta et al., 2020). These findings indicated that TMAO may be involved in the onset and development of kidney diseases as a biomarker of impaired kidney function. Reducing gut microbiota-dependent TMAO production may be a potential strategy for the treatment of DN. Despite the level of TMAO was proved to be positively associated with the progression of kidney disease, the TMAO receptor is still unknown, which may limit further investigation on TMAO targeted therapy. Researchers should pay attention to this field in the future studies.

### 4.4. Uremic toxins

Gut microbial metabolism derived UTs associated with kidney diseases are usually bound to protein, resulting in their little filtration by the glomerular barrier. Therefore, the accumulation of these UTs is the characteristic of decreased renal function. Relevant study has shown that the level of plasma UTs is elevated in patients with type 2 diabetes, which increase the risk of type 2 diabetes progressing to ESRD (Niewczas et al., 2014). Currently, IS, PS, pCS and p-cresyl glucuronide (pCG) are the mostly well-studied UTs in kidney diseases.

IS belongs to the indole class of UTs, which is originated from tryptophan metabolism by gut bacteria, including *Bacteroides ovatus*, *Clostridium limosum*, *Enterococcus faecalis* and *Escherichia coli* (Liu J. R. et al., 2021). IS, a ligand of aryl hydrocarbon receptor (AHR), plays a pivotal role in regulating podocyte function. The persistent activation of AHR by high-level IS can lead to the injury of podocytes and glomeruli (Tan et al., 2022). Relevant investigations suggested that the serum IS level is significantly increased in the patients with early stage of DN and correlated with renal function, inflammation and coronary atherosclerosis (Atoh et al., 2009; Chiu et al., 2010). Additionally, IS can also induce tubular cell death, oxidative stress, fibrosis and contribute to CKD progression. The low clearance rate of serum IS is significantly related to the mortality of advanced CKD patients. AST-120, a typical uremic toxin adsorbents, can reduce the serum level of IS and ameliorate the renal function by attenuating renal tubular injury and oxidative stress induced by IS (Cheng et al., 2020). PS is a uremic toxin synthesized from the gut microbial metabolites phenol, which enters the liver and undergoes a sulfate reaction. The intestinal microbes associated with phenol generation include Clostridiaceae, Enterococcaceae, Staphylococcaceae, Enterobacteriaceae, Bifidobacteriaceae and Bacteroidaceae (Smith and Macfarlane, 1996). The plasma PS level is associated with albumin-to-creatinine ratio (ACR) and 2-year progression of patients with DN. Moreover, administration of PS elicits albuminuria and podocyte injury in diabetic mice suggesting that PS, as a factor leading to kidney damage and proteinuria, may be a disease marker and therapeutic target for DN (Kikuchi et al., 2019). pCS and pCG are derived from tyrosine by intestinal bacteria through a series of deamination, transamination, and decarboxylation reactions (Gryp et al., 2017). Both pCS and pCG are markedly increased in the serum of patients with CKD, which contribute to CKD progression by disrupting renal tubule cell phenotype and functionality (Mutsaers et al., 2015; Wei et al., 2022). These findings suggested that UTs are not only the biomarkers for the occurrence of DN, but also the risk factors to promote the progression of DN. Reducing the generation of UTs by modulating gut microbiome can protect against DN. Nevertheless, the mechanism of UTs is not completely clear at present, and the approach of removing one UT may not be applicable to other UTs. The precise mechanisms of UTs in DN are still worth further exploration.

### 4.5. Hydrogen sulfide

H_2_S, an essential gaseous signaling molecule, is produced by the fermentation of sulfate or cysteine by sulfate-reducing bacteria in the gut lumen (Gui et al., 2021) and is involved in the regulation of multiple pathological processes, including inflammation (Wang L. et al., 2022), oxidative stress (Wang L. et al., 2022), endoplasmic reticulum stress (Zhao et al., 2022) and immunomodulation (Dilek et al., 2020). More recently, the function of H_2_S in renal physiology and disease states has attracted extensive attention. The potential roles of H_2_S in the regulation of eGFR, sodium absorption, renin release, and oxygen sensing in the renal system have been reported (Feng et al., 2022). Li et al. (2014) demonstrated that the level of plasma H_2_S is significantly lower in chronic hemodialysis patients with DN. The H_2_S deficiency contributes to the decline of kidney function and pathological status, with exogenous H_2_S can obviously improve DN (Zeng et al., 2016). For example, treatment with NaHS, a H_2_S donor, for 4 weeks significantly prevented the increase of blood glucose concentration and the body weight in STZ-induced diabetic rats. Meanwhile, NaHS significantly improved the renal function, and attenuated inflammatory cells infiltration, tubular atrophy and interstitial fibrosis in DN rats (Elbassuoni et al., 2020; Lodhi et al., 2021). Zhou et al. (2014) indicated that NaHS treatment reduced the release of inflammatory cytokines by inhibiting NF-κB activity in the kidney tissues of DN rats, suggesting that H_2_S can alleviate inflammatory response in DN. In addition, H_2_S can also ameliorate DN by inhibiting oxidative stress caused by hyperglycemia (Lodhi et al., 2021). Studies have found that intraperitoneal injection of NaHS alleviated DN by decreasing ROS generation, Nrf2 expression and MDA activity, and increasing SOD activity in the renal tissues of STZ-induced DN rats (Zhou et al., 2014; Ahmed et al., 2019). NaHS treatment increased the activity of sirtuin-1 (SIRT1) that exerts prominent renoprotective effect in various kidney diseases by regulating autophagy, lipid metabolism, apoptosis and sodium balance (Zhong et al., 2018; Ahmed et al., 2019). Particularly, extensive evidence has revealed that H_2_S can improve abnormal hemodynamics in DN. Exogenous H_2_S was proved to inhibit the activation of RAS in the kidney tissues of diabetic rats (Zhou et al., 2014). In diabetic mice, H_2_S significantly increased the blood flow, promoted the dilation of the renal peritubular capillary and improved diabetic renovascular remodeling (Yamamoto et al., 2013; Kundu et al., 2015). Moreover, exogenous H_2_S significantly lowered blood pressure and inhibited arterial medial calcification in STZ-induced DN rats (Ahmad et al., 2012; Wang F. Z. et al., 2021). These results emphasized the protective effects of H_2_S in DN. However, despite an increasing number of studies demonstrated that H_2_S, as a gut microbial metabolite, can effectively protect against DN through multiple routes, the clinical translation of these results is limited. The investigations on the biological effects of H_2_S in kidney contribute to understand the pathophysiology of DN and provide scientific basis for the development of new drugs based on H_2_S to treat DN.

## 5. Gut microbiota-targeted DN therapies

**Figure 4:**
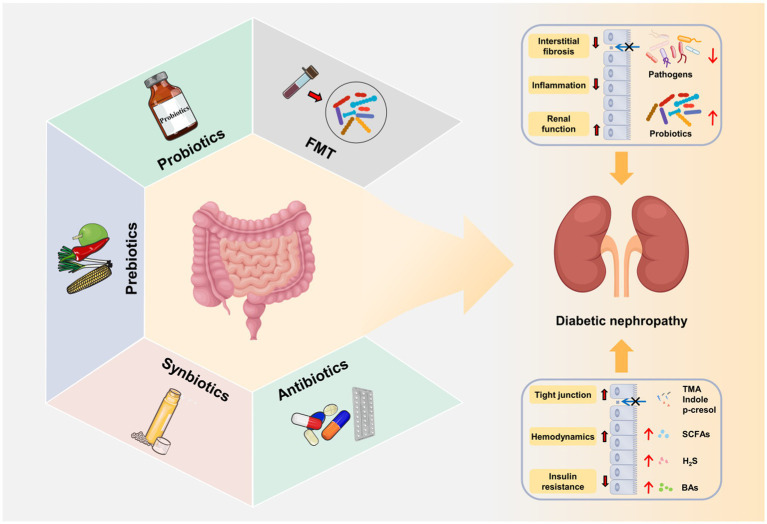
Gut microbiota-targeted DN therapies. Fecal microbiota transplantation (FMT), probiotics, prebiotics, synbiotics and antibiotics improve gut microbial dysbiosis, increase the release of beneficial metabolites (SCFAs, BAs, and H_2_S), decrease the production of harmful metabolites (LPS, TMAO, IS, and PCS) and repair intestinal barrier. The restoration of gut microbial microbial homeostasis further alleviates the progression of DN through gut–kidney axis.

Currently, the treatment of DN mainly includes lifestyle intervention, and the control of blood pressure, lipids, glucose and albuminuria (Wang J. M. et al., 2021). However, due to the complexity of the pathogenesis of DN, there is still a lack of standardized treatment. As discussed above, gut microbiota exerts a critical role in the onset and progression of DN (Table 3). The targeted intervention of gut microbiota by fecal microbiota transplantation (FMT), probiotics and prebiotics is expected to be a new strategy in the prevention and treatment of DN (Figure 4).

**Table 3:** Gut microbiota-targeted DN therapies.

Subjects	Therapeutic strategies	Types	Duration	Main effects	References
DN rats	FMT	Fecal microbiota suspension	3 days	Decreased serum acetate level and attenuated tubulointerstitial injury.	Hu et al. (2020)
DN rats	FMT	Fecal microbiota suspension	/	Improved podocyte insulin sensitivity and alleviated glomerular injury.	Lu J. et al. (2021)
Type 2 diabetic mice	FMT	Fecal microbiota suspension	8 weeks	Alleviated hyperglycemia, improved insulin resistance and injured islets and inhibited inflammation.	Wang et al. (2020)
DN mice	FMT	Fecal microbiota suspension	7 days	Improved renal function and inhibited inflammation.	Cai et al. (2020)
DN patients	Probiotics	*Lactobacillus plantarum* A7 (probiotic soy milk)	8 weeks	Improved renal function.	Abbasi et al. (2017)
DN patients	Probiotics	*Lactobacillus plantarum* A7 (probiotic soy milk)	8 weeks	Improved renal function and inflammatory factor.	Miraghajani et al. (2019)
DN patients	Probiotics	*Lactobacillus plantarum* A7 (probiotic soy milk)	8 weeks	Improved oxidative stress.	Miraghajani et al. (2017)
DN patients	Probiotics	*Bifidobacterium bifidum*, *Lactobacillus acidophilus* and *Streptococcus thermophilus* (probiotic supplements)	12 weeks	Ameliorate glycemic control.	Jiang et al. (2021)
DN patients	Probiotics	*Bacillus coagulans* T11 (probiotic honey)	12 weeks	Ameliorated insulin metabolism and dyslipidemia.	Arani et al. (2019)
DN patients	Probiotics	*Lactobacillus acidophilus*, *Bifidobacterium bifidum*, *Lactobacillus reuteri* and *Lactobacillus fermentum* (probiotic supplements)	12 weeks	Ameliorated glycemic control and markers of cardio-metabolic risk.	Mafi et al. (2018)
DN mice	Prebiotics	Inulin-type fructans	12 weeks	Improved renal function, glomerular injury and renal fibrosis and induced the expansion of acetate-generating bacteria.	Luo L. M. et al. (2022)
DN mice	Prebiotics	Dietary Fiber	9 weeks	Improving the renal function, kidney hypertrophy, glomerular injury and renal fibrosis, and promoted the enrichment of SCFA-producing bacteria.	Li Y. J. et al. (2020)
Diabetic rats	Prebiotics	FOS and XOS	6 weeks	Reduced hyperglycemia and cholesterol, improved renal function and renal injury, and restored the quantities of *bifidobacteria* and *lactobacilli*.	Gobinath et al. (2010)
Diabetic mice	Prebiotics	FOS	14 weeks	Decreased the endotoxemia, improved glucose tolerance and insulin resistance, inhibited inflammation and increased the quantities of *bifidobacteria.*	Cani et al. (2007)
CKD rats	Herbal medicines	*Rheum palmatum* L	4 weeks	Improved intestinal barrier, and alleviated renal fibrosis and inflammation, reduced the abundance of pernicious bacteria such as *Akkermansia*, *Methanosphaera* and *Clostridiaceae*, decreased the serum level of TMAO and increased the level of SCFAs.	Ji et al. (2020, 2021, 2022)
DN mice	Herbal medicines	QiDiTangShen granules	12 weeks	Alleviated renal injury, improved renal function, and restored intestinal barrier, decreased the abundance of *Lachnospiraceae_NK4A136_group*, *Lactobacillus* and *Bacteroides*, improved bile acid profiles.	Wei et al. (2021)
DN mice	Herbal medicines	Resveratrol	12 weeks	Improved renal function, glomerular lesion and inflammation, increased abundance of *Bacteroides*, *Alistipes* and *Parabacteroides.*	Cai et al. (2020)
Type 2 diabetic patients	Herbal medicines	Curcumin	15 days	Decreased the U-mAlb excretion and the level of plasma LPS. Increased the abundance of *Bacteroides*, *Bifidobacterium* and *Lactobacillus*, restored the epithelial barrier and inhibited inflammatory response.	Yang et al. (2015)
CKD patients	Synbiotics	*Lactobacillus*, *Bifidobacteria* and *Streptococcus* (probiotic capsule), inulin, FOS and GOS	6 weeks	Reduced serum PCS and improved gut microbiome.	Rossi et al. (2016)
CKD rats	Synbiotics	*Lactobacillus*, *Bifidobacterium*, *Streptococcus* (probiotic supplements) and inulin	5 weeks or 10 weeks	Reduced the indole-generating bacteria of *Clostridium* and restored gut microbiota diversity.	Yang et al. (2021)
DN rats	Antibiotics	Ampicillin, neomycin, vancomycin and amphotericin B (antibiotic cocktail)	8 weeks	Eliminate the majority of the gut microbiota, reduced plasma acetate level and alleviated kidney injury.	Lu et al. (2020)
DN rats	Antibiotics	Vancomycin, neomycin, metronidazole, amphotericin B, ampicillin (antibiotic cocktail)	8 weeks	Attenuated albuminuria and tubulointerstitial injury, and reduced serum acetate levels.	Hu et al. (2020)
DN mice	Diet	Dietary fiber	12 weeks	Improved renal function, glomerular injury and renal fibrosis, promoted the expansion of SCFAs-producing bacteria, and reduced inflammatory cytokines.	Li Y. J. et al. (2020), Drake et al. (2022)
DN rats	Diet	Mulberry leaf tea	13 weeks	Ameliorated hyperglycemia and renal injury, improved the community structure of gut microbiota.	Sheng et al. (2018)
Type 2 diabetic mice	Exercise	Running	6 weeks	Increased the amount of *Firmicutes* and decreased the amount of *Bacteroides/Prevotella.*	Lambert et al. (2015)
Type 2 diabetic mice	Exercise	Swimming	8 weeks	Increased the abundance of *Bacteroides* and the level of SCFAs.	Yang L. et al. (2020)
Type 2 diabetic patients	Exercise	/	6 months	Improved glycemia, intestinal barrier and systemic inflammation.	Pasini et al. (2019)

### 5.1. Fecal microbiota transplantation

Fecal microbiota transplantation (FMT) is an emerging therapeutic method that transplants the functional microbiota from healthy donors into the gastrointestinal tract of patients with pathological microbiota (Waller et al., 2022). As a promising microbiome-based method, FMT is mainly used to treat intestinal diseases (Waller et al., 2022) and metabolic diseases (Qu et al., 2022). To date, the investigations of FMT in DN are relatively lacking, although thousands of patients worldwide have received FMT therapy.

In preclinical studies, FMT was administrated from healthy control rats to STZ-induced diabetic rats. The results showed that FMT significantly decreased serum acetate level, restored the cholesterol homeostasis, improved podocyte insulin sensitivity, and attenuated tubulointerstitial and glomerular injury (Hu et al., 2020; Lu J. et al., 2021). Consistently, FMT was found to alleviated hyperglycemia, improved insulin resistance and injured islets and inhibited inflammation in STZ-induced type 2 diabetes (Wang et al., 2020). Moreover, another study reported that resveratrol, a polyphenolic compound, can improve DN by restoring gut microbiota homeostasis. FMT from resveratrol-treated mice to diabetic mice significantly inhibited inflammation, and improved renal function and intestinal characteristics (Cai et al., 2020). Most recently, further study confirmed the safety and efficacy of FMT method in the treatment of DN (Bastos et al., 2022). Of note, DN mice received FMT from mice with severe proteinuria showed higher concentrations of LPS, TMAO and 24-h UP, lower levels of propionic acid and butyric acid, and different gut microbiome composition compared to those receiving FMT from mice with mild proteinuria, which further suggested the important role of gut microbiome in the development of DN and the possibility of FMT in the treatment of DN (Li Y. et al., 2020).

Given that FMT technique can potently ameliorate DN by regulating the dysregulated gut–kidney axis, it has attracted widespread attentions as a microbiota-based intervention in DN. Nevertheless, the applications of FMT in patients with DN are small-scale, because the fecal extract may transmit occult infections. Hence, there is an urgent need to establish rigorous donor screening guidelines to reduce the risk of infection. In addition, more clinical studies are needed to determine some factors that affect FMT, such as the sample preparation, storage, dose and delivery route, to make this process standard.

### 5.2. Probiotics

Probiotics are live non-pathogenic microorganisms that colonize the gastrointestinal tract and confer health benefits when ingested in adequate amounts (Singh et al., 2022). An increasing number of investigations have demonstrated that probiotics exhibit promising properties in maintaining intestinal homeostasis, enhancing nutrient assimilation and promoting immunity (Wu T. Y. et al., 2022). Currently, the most widely used probiotics include *Lactobacillus* and *Bifidobacterium* (Zheng et al., 2021). In the past few years, mounting evidence has reported the renoprotective effect of probiotics by regulating gut–kidney axis (Koppe et al., 2015).

A randomized controlled clinical trial demonstrated that soy milk containing *Lactobacillus plantarum* A7 significantly reduced the albuminuria, serum creatinine and improved eGFR in patients with DN compared with the conventional soy milk, suggesting that probiotic *Lactobacillus plantarum* A7 can improve the renal function of DN patients (Abbasi et al., 2017). Consistently, probiotic *Lactobacillus plantarum* A7 was reported to improve inflammatory and oxidative stress factors, which further indicated the beneficial effect of probiotic *Lactobacillus plantarum* A7 in DN patients (Miraghajani et al., 2017, 2019). In addition, other investigations also indicated that probiotic can ameliorate hyperglycemia and dyslipidemia in DN patients. Jiang et al. reported that probiotic supplements (*Bifidobacterium bifidum*, *Lactobacillus acidophilus* and *Streptococcus thermophilus*) significantly reduced the level of FBG and HbA1c in DN patients (Jiang et al., 2021). The honey containing probiotic *Bacillus coagulans* T11 decreased the radio of total-/HDL-cholesterol (Arani et al., 2019). Additionally, DN is usually accompanied by chronic hyperglycemia resulting from the deficiency of insulin sensitivity. A randomized clinical study demonstrated that a mixture of *Lactobacillus acidophilus*, *Bifidobacterium bifidum*, *Lactobacillus reuteri* and *Lactobacillus fermentum* effectively increased insulin sensitivity and ameliorated glycemic control in patients with DN (Mafi et al., 2018), the similar effects were observed by probiotic *Bacillus coagulans* T11honey consumption in DN patients (Arani et al., 2019).

Such findings highlighted the potential of probiotics as an auxiliary measure for the treatment of DN. Overall, these investigations indicated that probiotics may exert beneficial role for DN patients by improving renal function, inflammation and oxidative stress, and controlling glycemia and dyslipidemia. Although probiotics possess a broad prospect in the treatment of DN, gaps still exist in the clinical applications of probiotics. For instance, few subsequent studies have shown that the administration of probiotics can maintain the long-term colonization of the gut microbiota DN patients. Probiotics may also have potential risks for patients with immunodeficiency as opportunistic pathogens (Doron and Snydman, 2015). Additionally, the selection and dosage of probiotics depend on the specific intestinal environment. Related investigations on this field are scarce.

### 5.3. Prebiotics

Prebiotics are described as substrates that exhibit beneficial health effects to the host when selectively used by microorganisms (Gibson et al., 2017). The most well-known prebiotics mainly include inulin, dextran, pectins, lactulose, fructooligosaccharides (FOS) and galactooligosaccharides (GOS; Zakrzewska et al., 2022). It has been reported that prebiotics play a critical role in the modulating immune system (Pujari and Banerjee, 2021) and maintaining gastrointestinal function (Holscher, 2017), of which are associated with inflammation and gut microbiota dysbiosis in DN (Zhang L. L. et al., 2022).

A recent animal experiment demonstrated that inulin-type fructans (ITFs) supplementation induced the expansion of acetate-generating bacteria, especially the genera *Akkermansia* and *Candidatus Saccharimonas*, and improved renal function, glomerular injury and renal fibrosis in db/db mice (Luo L. M. et al., 2022). These results are consistent with those of diabetic mice treated with a high-fiber diet. Compared with diabetic mice on a diet, dietary fiber treatment inhibited development of diabetes to DN by improving the renal function and pathological injury. Dietary fiber not only restored the gut microbial community, but also increased the concentration of SCFAs by promoting the enrichment of SCFA-producing bacteria, such as the genera *Prevotella* and *Bifidobacterium* (Li Y. J. et al., 2020). These studies suggested that prebiotics exert a renoprotective function through SCFAs in DN. FOS has been shown to ameliorate DN by improving gut dysbiosis and inflammation (Pengrattanachot et al., 2022). Treatment with FOS decreased the endotoxemia, improved glucose tolerance and insulin resistance, and inhibited inflammation in high-fat-diet-induced diabetic mice (Cani et al., 2007). Additionally, Gobinath et al. used FOS and xylo-oligosaccharides (XOS) as a diet supplement for STZ-induced diabetic rats. As expected, supplementation of FOS and XOS restored the quantities of *bifidobacteria* and *lactobacilli*, which led to the improved renal function, reduced lipid accumulation and attenuated renal injury (Gobinath et al., 2010).

Taken together, these results indicated that as a functional food, prebiotics can delay the progression of DN by altering microbial compositions and functions. Nevertheless, most of the current studies on the intervention of DN by prebiotics were performed in animals, and thereby clinical studies are needed to confirm the practical application of prebiotics in humans. Moreover, the specific mechanism of prebiotics to improve DN requires further study, as the causes of DN are diverse. In recent decades, prebiotics have attracted extensive attention due to their safety, effectiveness and non-toxic side effects. Based on the prebiotics, it is of great significance to clarify the mechanism and combine the dietary intervention with traditional treatments for DN therapies.

### 5.4. Herbal medicines and their active ingredients

As well known, herbal medicine exhibits robust therapeutic efficacy for kidney diseases. In recent years, some studies have proved that herbal medicine can improve DN by regulating gut microbiota dysbiosis. Ji et al. found that *Rheum palmatum* L. can improve intestinal barrier, and alleviate renal fibrosis and inflammation by reducing the abundance of pernicious bacteria such as *Akkermansia*, *Methanosphaera* and *Clostridiaceae* in CKD rats (Ji et al., 2020). Meanwhile, they found that *Rheum palmatum* L. treatment decreased the serum level of TMAO and increased the level of SCFAs (butanoic acid and isobutyric acid), which further confirmed the renoprotective role of *Rheum palmatum* L. (Ji et al., 2021, 2022). Moreover, many traditional Chinese herbal formulas have also been reported to exert good efficacy on the treatment of DN, and the underlying mechanism involves the regulation of gut microbiota dysbiosis. For example, treatment with QiDiTangShen granules significantly alleviated renal injury, improved renal function, and restored intestinal barrier in DN mice. Mechanistically, QiDiTangShen granules decreased the abundance of *Lachnospiraceae_NK4A136_group*, *Lactobacillus* and *Bacteroides*, which were positively correlated with renal injury indicators. Further study demonstrated that QiDiTangShen granules exert the renoprotective function through gut microbiota-bile acid axis (Wei et al., 2021). Other traditional Chinese herbal formulas such as Qing-Re-Xiao-Zheng formula (Gao et al., 2021), Tangshen formula (Zhao et al., 2020) and Zicuiyin decoction (Liu J. et al., 2022) were also found to exert anti-DN role by modulating gut microbiota dysbiosis.

What is more, many active compounds isolated from herbal medicines also possess excellent anti-DN properties. For example, Resveratrol (10 mg/kg b. w.) administration for 12 weeks significantly improved renal function, glomerular lesion and inflammation in db/db mice, along with the increased abundance of *Bacteroides*, *Alistipes* and *Parabacteroides*, which exhibit anti anti-inflammatory properties (Cai et al., 2020). Curcumin, a major active component of turmeric, markedly decreased the urinary micro-albumin (U-mAlb) excretion and the level of plasma LPS in patients with T2DM (500 mg/d for 15 days). The potential mechanism may be that curcumin increased the abundance of *Bacteroides*, *Bifidobacterium* and *Lactobacillus*, which restored the epithelial barrier and inhibited inflammatory response induced by LPS (Yang et al., 2015). In addition, some natural polysaccharides, such as Moutan Cortex polysaccharides (Zhang M. et al., 2022) and *Bupleurum* polysaccharides (Feng et al., 2019) were also reported to amelirate DN *via* modulating gut microbiota composition through gut–kidney axis. These investigations have revealed the potential role of gut microbiota in the treatment of DN with herbal medicines. However, there is still relatively little research in this field. Future studies should focus on the underlying mechanisms by which herbal medicines exert their renoprotective function through gut–kidney axis. Clarifying the roles of gut microbiota in the treatment of kidney diseases by herbal medicines will provide new strategies for the diagnosis and therapy of DN.

### 5.5. Others

Except for the three main approaches mentioned above, there are other gut microbiota-targeted DN therapies, including synbiotics, antibiotics, diet and exercise. Synbiotics are defined as the mixtures of probiotics and prebiotics, which can promote the survival of probiotic in the intestinal tract (Jiang et al., 2022). Studies have shown that the combination of probiotics and prebiotics can exert a synergistic effect, which is more efficient compared to probiotics or prebiotics alone (Gibson et al., 2017). In a randomized, double-blind and placebo controlled trial, the symbiotic effect of the probiotic (*Bifidobacteria*, *Lactobacillus* and *Streptococcus*) and the prebiotics (FOS, GOS and inulin) ameliorated renal failure by reducing serum PCS and improving gut microbiome composition in CKD patients (Rossi et al., 2016). In adenine-induced CKD rats, the treatment of synbiotics containing *Bifidobacteria*, *Lactobacillus*, *Streptococcus* and inulin caused a reduction in the indole-generating bacteria of *Clostridium* and the quantity of fecal indole. Additionally, synbiotics treatment restored gut microbiota diversity in CKD rats (Yang et al., 2021), indicating that synbiotics exert a renoprotective effect by improving gut indole load and gut microbiota dysbiosis. Antibiotics are also widely used in the treatment of kidney diseases by targeting gut microbiota (Aloy et al., 2020). Lu et al. (2020) demonstrated that broad-spectrum antibiotics administration for 8 weeks eliminated the majority of the gut microbiota, reduced plasma acetate level and suppressed the activation of intrarenal RAS activation, and thereby alleviated kidney injury in STZ-induced DN rats. Similarly, the depletion of intestinal microflora by antibiotics was reported to ameliorate the dysregulation of cholesterol homeostasis in the tubulointerstitium of DN rats (Hu et al., 2020). However, the abuse of antibiotics may also lead to the kidney injury and the invasion of antibiotic-associated pathogens (Kamada et al., 2013). Thus, the antibiotic therapy needs further research to ensure its safety. In addition, lifestyle intervention, such as dietotherapy and exercise training, is also a common strategy for DN therapy. Dietary fiber was proved to attenuate the progression of DN by promoting the expansion of SCFAs-producing bacteria, such as *Prevotella* and *Bifidobacterium*, which can inhibit inflammation and oxidative stress (Li Y. J. et al., 2020; Drake et al., 2022). In STZ-induced DN rats, Mulberry leaf tea markedly ameliorated hyperglycemia and renal injury by restoring the abundance of *Adlercreutzia*, *Anaerofustis*, *Coprococcus*, *Dorea*, *Roseburia*, *Oscillospira*, *Phascolarctobacterium*, *Coprobacillus*, *Sutterella* and *RF39*, which indicated that Mulberry leaf tea can be used as a dietotherapy for DN therapy through gut–kidney axis (Sheng et al., 2018). Exercise training has been reported to be a novel nonpharmacological approach in the modulation of gut microbiota, which could benefit individuals by restoring gut microbiota composition and function (Wegierska et al., 2022). For example, Lambert et al. demonstrated that exercise training for 6 weeks has significantly increased the amount of Firmicutes and decreased the amount of *Bacteroides/Prevotella* spp. in type 2 diabetic mice (Lambert et al., 2015). Further, another similar investigation indicated that aerobic exercise for 8 weeks reversed the decreased abundance of *Bacteroides*, and increased the level of SCFAs in type 2 diabetic mice (Yang L. et al., 2020). In a clinical trial, the intestinal barrier and systemic inflammation of patients with type 2 diabetes were significantly improved after chronic exercise for 6 months, suggesting that exercise might be a potential therapeutic strategy for DN *via* gut–kidney axis (Pasini et al., 2019). Unfortunately, there is no direct evidence that exercise training can ameliorate DN by modifying gut microbiota. Currently, the exercise training regimen for DN therapy is still in the initial stage (Amaral et al., 2020). Thus, researchers need to do considerable work to build appropriate, feasible and scientific exercise program for the patients with DN.

## 6. Summary and prospects

This review summarized the specific alterations of gut microbiome in DN and elucidated the correlations between gut microbiota and DN phenotypes, and it will provide a new insight for the further investigations of gut microbiota in DN. Nevertheless, gaps still exist in these previous studies. First of all, these investigations were cross-sectional, and failed to reflect the alterations and correlations in different stages of DN progression. The sample size of some clinical studies are insufficient to reveal the real relationships between gut microbiota and DN. Secondly, the causal relationship between gut microbial dysbiosis and DN onset is unclear. The intestinal microbial dysbiosis associated with DN may occur in small intestine (Shi et al., 2023), while most of these studies on gut microbiota are based on fecal sample. Finally, gut microbiota can be influenced by many factors, such as diet, age, gender and medications. These factors should be taken into account in future studies to better clarify the associations between gut microbiota and DN.

Gut microbial metabolites, including SCFAs, BAs, TMAO, UTs and H_2_S, were also discussed in detail. As a bridge between gut microbiota and host, these metabolites play essential roles in the onset and development of DN. The exact mechanisms by which microbial metabolites modulate DN require further investigation in animal models and a large patient cohorts. Moreover, researchers should focus on the route, frequency and concentration of administration for some beneficial metabolites, such as SCFAs and H_2_S. As for those metabolites that contribute to the pathophysiology of the disease, such as UTs and TMAO, future investigations should focus on the development of inhibitors. Exploiting related enzymes that generate these harmful metabolites may be a promising strategy.

DN results in high morbidity and mortality worldwide and effective therapeutic strategies for DN are urgently needed. Mounting studies have demonstrated that DN is accompanied by gut microbial dysbiosis and alterations to microbial metabolites, suggesting that gut microbiota-targeted therapies may be an effective strategy against DN. Rational utilization of probiotics and prebiotics, as well as FMT method contribute to restoring intestinal flora homeostasis, and thereby retarding DN progression. However, many current studies, especially FMT technique, are only limited to preclinical animal studies, and more reliable clinical trials are needed to confirm the results. Additionally, the combination of gut microbiota-targeted therapies with medications may has a better effect, while the investigation in this field is scarce. Therefore, researchers need to do considerable work to bridging these gaps.

## Author contributions

JY: study concepts and design. HZ, C-EY, TL, and M-XZ: literature search. HZ, C-EY, YN, and MW: manuscript preparation and revision. All authors contributed to the article and approved the submitted version.

## Funding

This work was supported by National Science Basic Research Program of Shaanxi and Basic Research Program of Xi’an Municipal Health Commission (Nos. 2022JQ-920, 2022yb41, 82204745, 2023qn22).

## Conflict of interest

The authors declare that the research was conducted in the absence of any commercial or financial relationships that could be construed as a potential conflict of interest.

## Publisher’s note

All claims expressed in this article are solely those of the authors and do not necessarily represent those of their affiliated organizations, or those of the publisher, the editors and the reviewers. Any product that may be evaluated in this article, or claim that may be made by its manufacturer, is not guaranteed or endorsed by the publisher.

## Abbreviations

AGEs: advanced glycation end products
AHR: aryl hydrocarbon receptor
ANG-II: angiotensin II
BAs: bile acids
CKD: chronic kidney disease
DG: diacylglycerol
DN: diabetic nephropathy
eGFR: estimated glomerular filtration rate
ESRD: end-stage renal disease
ET-1: endothelin-1
F/B: *Firmicutes/Bacteroidetes*
FBG: fasting blood glucose
FMT: fecal microbiota transplantation
FOS: fructooligosaccharides
FXR: farnesoid X receptor
GOS: galactooligosaccharides
GPCRs: G-protein-coupled receptors
HbA1c: glycosylated hemoglobin
HDAC: histone acetylation
H_2_S: hydrogen sulfide
24-h UP: 24-h urine protein
IL-1: interleukin-1
IL-6: interleukin-1
IS: indoxyl sulfate
LPS: lipopolysaccharide
MDA: Malondialdehyde
NaB: sodium butyrate
NADPH: nicotinamide adenine dinucleotide phosphate
NF-κ B: nuclear factor-κB
NLRP3: nod-like receptor (NLR) family pyrin domain containing 3
Nrf2: nuclear factor E2 related factor 2
PCS: p-cresyl sulfate
pCG: p-cresyl glucuronide
PKC: protein kinase C
PS: phenyl sulfate
RAGE: receptor for advanced glycation end products
RAS: renin-angiotensin system
ROS: reactive oxygen species
SCFAs: short-chain fatty acid
SCR: serum creatinine
SOD: superoxide dismutase
STZ: streptozotocin
TC: total cholesterol
TG: triglyceride
TGF-β1: transforming growth factor-β1
TGR5: takeda G protein-coupled receptor 5
TLR: toll like receptors
TMAO: trimethylamine-N-oxide
TNF-α: tumor necrosis factor-α
UACR: urinary albumin-to-creatinine ratio
UTs: uremic toxins
XOS: xylo-oligosaccharides

## References

[ref1] Abbasi B. Ghiasvand R. Mirlohi M. (2017). Kidney function improvement by soy milk containing *Lactobacillus plantarum* A7 in type 2 diabetic patients with nephropathy a double-blinded randomized controlled trial. Iran. J. Kidney Dis. 11, 36–43. 28174351

[ref2] Adachi K. Sugiyama T. Yamaguchi Y. Tamura Y. Izawa S. Hijikata Y.. (2019). Gut microbiota disorders cause type 2 diabetes mellitus and homeostatic disturbances in gut-related metabolism in Japanese subjects. J. Clin. Biochem. Nutr. 64, 231–238. doi: 10.3164/jcbn.18-101, PMID: 31138957 PMC6529700

[ref3] Ahmad F. U. D. Sattar M. A. Rathore H. A. Abdullah M. H. Tan S. Abdullah N. A.. (2012). Exogenous hydrogen sulfide (H_2_S) reduces blood pressure and prevents the progression of diabetic nephropathy in spontaneously hypertensive rats. Ren. Fail. 34, 203–210. doi: 10.3109/0886022x.2011.643365, PMID: 22229751

[ref4] Ahmed H. H. Taha F. M. Omar H. S. Elwi H. M. Abdelnasser M. (2019). Hydrogen sulfide modulates SIRT1 and suppresses oxidative stress in diabetic nephropathy. Mol. Cell. Biochem. 457, 1–9. doi: 10.1007/s11010-019-03506-x 30778838

[ref5] al-Obaide M. Singh R. Datta P. Rewers-Felkins K. A. Salguero M. V. al-Obaidi I.. (2017). Gut microbiota-dependent trimethylamine-N-oxide and serum biomarkers in patients with T2DM and advanced CKD. J. Clin. Med. 6:86. doi: 10.3390/jcm6090086, PMID: 28925931 PMC5615279

[ref6] Aloy B. Launay-Vacher V. Bleibtreu A. Bortolotti P. Faure E. Filali A.. (2020). Antibiotics and chronic kidney disease: dose adjustment update for infectious disease clinical practice. Med. Mal. Infect. 50, 323–331. doi: 10.1016/j.medmal.2019.06.010, PMID: 31326299

[ref7] Amaral L. S. D. Souza C. S. Lima H. N. Soares T. D. (2020). Influence of exercise training on diabetic kidney disease: a brief physiological approach. Exp. Biol. Med. 245, 1142–1154. doi: 10.1177/1535370220928986 PMC7400720 32486850

[ref8] Arani N. M. Emam-Djomeh Z. Tavakolipour H. Sharafati-Chaleshtori R. Soleimani A. Asemi Z. (2019). The effects of probiotic honey consumption on metabolic status in patients with diabetic nephropathy: a randomized, double-blind, controlled trial. Probiotics Antimicro. 11, 1195–1201. doi: 10.1007/s12602-018-9468-x 30218286

[ref9] Atoh K. Itoh H. Haneda M. (2009). Serum indoxyl sulfate levels in patients with diabetic nephropathy: relation to renal function. Diabetes Res. Clin. Pract. 83, 220–226. doi: 10.1016/j.diabres.2008.09.053 19027976

[ref10] Bai Y. F. Li P. Liu J. A. Zhang L. Cui S. Y. Wei C. T.. (2022). Renal primary cilia lengthen in the progression of diabetic kidney disease. Front. Endocrinol. 13:984452. doi: 10.3389/fendo.2022.984452, PMID: 36465609 PMC9713695

[ref11] Bastos R. M. C. Simplício-Filho A. Sávio-Silva C. Oliveira L. F. V. Cruz G. N. F. Sousa E. H.. (2022). Fecal microbiota transplant in a pre-clinical model of type 2 diabetes mellitus, obesity and diabetic kidney disease. Int. J. Mol. Sci. 23:3842. doi: 10.3390/ijms23073842, PMID: 35409202 PMC8998923

[ref12] Bertolini A. Fiorotto R. Strazzabosco M. (2022). Bile acids and their receptors: modulators and therapeutic targets in liver inflammation. Semin. Immunopathol. 44, 547–564. doi: 10.1007/s00281-022-00935-7 35415765 PMC9256560

[ref13] Bui T. M. Wiesolek H. L. Sumagin R. (2020). ICAM-1: a master regulator of cellular responses in inflammation, injury resolution, and tumorigenesis. J. Leukoc. Biol. 108, 787–799. doi: 10.1002/jlb.2mr0220-549r 32182390 PMC7977775

[ref14] Cai J. Rimal B. Jiang C. Chiang J. Y. L. Patterson A. D. (2022). Bile acid metabolism and signaling, the microbiota, and metabolic disease. Pharmacol. Ther. 237:108238. doi: 10.1016/j.pharmthera.2022.108238 35792223

[ref15] Cai T. T. Ye X. L. Li R. R. Chen H. Wang Y. Y. Yong H. J.. (2020). Resveratrol modulates the gut microbiota and inflammation to protect against diabetic nephropathy in mice. Front. Pharmacol. 11:1249. doi: 10.3389/fphar.2020.01249, PMID: 32973502 PMC7466761

[ref16] Cani P. D. Neyrinck A. M. Fava F. Knauf C. Burcelin R. G. Tuohy K. M.. (2007). Selective increases of bifidobacteria in gut microflora improve high-fat-diet-induced diabetes in mice through a mechanism associated with endotoxaemia. Diabetologia 50, 2374–2383. doi: 10.1007/s00125-007-0791-0, PMID: 17823788

[ref17] Chang Y. C. Chu Y. H. Wang C. C. Wang C. H. Tain Y. L. Yang H. W. (2021). Rapid detection of gut microbial metabolite trimethylamine N-oxide for chronic kidney disease prevention. Biosensors-Basel 11:339. doi: 10.3390/bios11090339 34562929 PMC8469701

[ref18] Chen X. Dai W. N. Li H. Yan Z. Liu Z. W. He L. Y. (2023). Targeted drug delivery strategy: a bridge to the therapy of diabetic kidney disease. Drug Deliv. 30:2160518. doi: 10.1080/10717544.2022.2160518 36576203 PMC9809356

[ref19] Chen J. H. Liu Q. H. He J. H. Li Y. P. (2022). Immune responses in diabetic nephropathy: pathogenic mechanisms and therapeutic target. Front. Immunol. 13:958790. doi: 10.3389/fimmu.2022.958790 36045667 PMC9420855

[ref20] Chen Q. Ren D. W. Wu J. Q. Yu H. Y. Chen X. P. Wang J.. (2021). Shenyan Kangfu tablet alleviates diabetic kidney disease through attenuating inflammation and modulating the gut microbiota. J. Nat. Med. 75, 84–98. doi: 10.1007/s11418-020-01452-3, PMID: 32997272

[ref21] Chen W. H. Zhang M. J. Guo Y. Wang Z. Liu Q. Q. Yan R. Z.. (2021). The profile and function of gut microbiota in diabetic nephropathy. Diabetes Metab. Syndr. Obes. 14, 4283–4296. doi: 10.2147/dmso.s320169, PMID: 34703261 PMC8541750

[ref22] Cheng T. H. Ma M. C. Liao M. T. Zheng C. M. Lu K. C. Liao C. H.. (2020). Indoxyl sulfate, a tubular toxin, contributes to the development of chronic kidney disease. Toxins 12, 684. doi: 10.3390/toxins12110684, PMID: 33138205 PMC7693919

[ref23] Chiu C.-A. Lu L.-F. Yu T.-H. Hung W.-C. Chung F.-M. Tsai I. T.. (2010). Increased levels of Total P-Cresylsulphate and Indoxyl Sul-phate are associated with coronary artery disease in patients with diabetic nephropathy. Rev. Diabet. Stud. 7, 275–284. doi: 10.1900/rds.2010.7.275, PMID: 21713315 PMC3143542

[ref24] Dang G. Q. Wu W. D. Zhang H. F. Everaert N. (2021). A new paradigm for a new simple chemical: butyrate and immune regulation. Food Funct. 12, 12181–12193. doi: 10.1039/d1fo02116h 34752597

[ref25] DeFronzo R. A. Reeves W. B. Awad A. S. (2021). Pathophysiology of diabetic kidney disease: impact of SGLT2 inhibitors. Nat. Rev. Nephrol. 17, 319–334. doi: 10.1038/s41581-021-00393-8 33547417

[ref26] Deng L. Yang Y. Xu G. S. (2022). Empagliflozin ameliorates type 2 diabetes mellitus-related diabetic nephropathy via altering the gut microbiota. BBA-Mol. Cell Biol. L. 1867:159234. doi: 10.1016/j.bbalip.2022.159234 36185030

[ref27] Dilek N. Papapetropoulos A. Toliver-Kinsky T. Szabo C. (2020). Hydrogen sulfide: an endogenous regulator of the immune system. Pharmacol. Res. 161:105119. doi: 10.1016/j.phrs.2020.105119 32781284

[ref28] Ding L. L. Yang Q. L. Zhang E. Y. Wang Y. M. Sun S. M. Yang Y. B.. (2021). Notoginsenoside Ft1 acts as a TGR5 agonist but FXR antagonist to alleviate high fat diet-induced obesity and insulin resistance in mice. Acta Pharm. Sin. B 11, 1541–1554. doi: 10.1016/j.apsb.2021.03.038, PMID: 34221867 PMC8245856

[ref29] Dong W. P. Jia Y. Liu X. X. Zhang H. Li T. Huang W. L.. (2017). Sodium butyrate activates NRF2 to ameliorate diabetic nephropathy possibly via inhibition of HDAC. J. Endocrinol. 232, 71–83. doi: 10.1530/joe-16-0322, PMID: 27799462

[ref30] Doron S. Snydman D. R. (2015). Risk and safety of probiotics. Clin. Infect. Dis. 60, S129–S134. doi: 10.1093/cid/civ085 25922398 PMC4490230

[ref31] Drake A. M. Coughlan M. T. Christophersen C. T. Snelson M. (2022). Resistant starch as a dietary intervention to limit the progression of diabetic kidney disease. Nutrients 14:4547. doi: 10.3390/nu14214547 36364808 PMC9656781

[ref32] du X. Liu J. Xue Y. Kong X. Y. Lv C. X. Li Z. Q.. (2021). Alteration of gut microbial profile in patients with diabetic nephropathy. Endocrine 73, 71–84. doi: 10.1007/s12020-021-02721-1, PMID: 33905112

[ref33] Du Y. Yang Y. T. Tang G. Jia J. S. Zhu N. Yuan W. J. (2020). Butyrate alleviates diabetic kidney disease by mediating the miR-7a-5p/P311/TGF-β1 pathway. FASEB J. 34, 10462–10475. doi: 10.1096/fj.202000431R 32539181

[ref34] Eckburg P. B. Bik E. M. Bernstein C. N. Purdom E. Dethlefsen L. Sargent M.. (2005). Diversity of the human intestinal microbial flora. Science 308, 1635–1638. doi: 10.1126/science.1110591, PMID: 15831718 PMC1395357

[ref35] Elbassuoni E. A. Aziz N. M. Habeeb W. N. (2020). The role of activation of K-ATP channels on hydrogen sulfide induced renoprotective effect on diabetic nephropathy. J. Cell. Physiol. 235, 5223–5228. doi: 10.1002/jcp.29403 31774182

[ref36] Ergul A. (2011). Endothelin-1 and diabetic complications: focus on the vasculature. Pharmacol. Res. 63, 477–482. doi: 10.1016/j.phrs.2011.01.012 21292003 PMC7383935

[ref37] Fang Q. Zheng B. J. Liu N. Liu J. F. Liu W. H. Huang X. Y.. (2021). Trimethylamine N-oxide exacerbates renal inflammation and fibrosis in rats with diabetic kidney disease. Front. Physiol. 12:682482. doi: 10.3389/fphys.2021.682482, PMID: 34220546 PMC8243655

[ref38] Feng J. A. Lu X. X. Li H. Wang S. X. (2022). The roles of hydrogen sulfide in renal physiology and disease states. Ren. Fail. 44, 1289–1308. doi: 10.1080/0886022x.2022.2107936 35930288 PMC9359156

[ref39] Feng Y. Weng H. Ling L. Zeng T. Zhang Y. Chen D.. (2019). Modulating the gut microbiota and inflammation is involved in the effect of Bupleurum polysaccharides against diabetic nephropathy in mice. Int. J. Biol. Macromol. 132, 1001–1011. doi: 10.1016/j.ijbiomac.2019.03.242, PMID: 30946910

[ref40] Fernandes M. R. Aggarwal P. Costa R. G. F. Cole A. M. Trinchieri G. (2022). Targeting the gut microbiota for cancer therapy. Nat. Rev. Cancer 22, 703–722. doi: 10.1038/s41568-022-00513-x 36253536

[ref41] Forst T. Mathieu C. Giorgino F. Wheeler D. C. Papanas N. Schmieder R. E.. (2022). New strategies to improve clinical outcomes for diabetic kidney disease. BMC Med. 20:337. doi: 10.1186/s12916-022-02539-2, PMID: 36210442 PMC9548386

[ref42] Fuchs C. D. Dixon E. D. Hendrikx T. Mlitz V. Wahlström A. Ståhlman M.. (2022). Tetrahydroxylated bile acids improve cholestatic liver and bile duct injury in the Mdr2(−/−) mouse model of sclerosing cholangitis via immunomodulatory effects. Hepatol. Commun. 6, 2368–2378. doi: 10.1002/hep4.1998, PMID: 35691019 PMC9426398

[ref43] Gao Y. B. Yang R. B. Guo L. Wang Y. X. Liu W. J. Ai S. N.. (2021). Qing-re-Xiao-Zheng formula modulates gut microbiota and inhibits inflammation in mice with diabetic kidney disease. Front. Med. 8:719905. doi: 10.3389/fmed.2021.719950, PMID: 34604258 PMC8481597

[ref44] Gibson G. R. Hutkins R. Sanders M. E. Prescott S. L. Reimer R. A. Salminen S. J.. (2017). Expert consensus document: the international scientific association for probiotics and prebiotics (ISAPP) consensus statement on the definition and scope of prebiotics. Nat. Clin. Pract. Gastroenterol. Hepatol. 14, 491–502. doi: 10.1038/nrgastro.2017.75, PMID: 28611480

[ref45] Gilbert J. A. Blaser M. J. Caporaso J. G. Jansson J. K. Lynch S. V. Knight R. (2018). Current understanding of the human microbiome. Nat. Med. 24, 392–400. doi: 10.1038/nm.4517 29634682 PMC7043356

[ref46] Gobinath D. Madhu A. Prashant G. Srinivasan K. Prapulla S. (2010). Beneficial effect of xylo-oligosaccharides and fructo-oligosaccharides in streptozotocin-induced diabetic rats. Brit. J. Nutr. 104, 40–47. doi: 10.1017/s0007114510000243 20187988

[ref47] Gradisteanu G. P. Stoica R. A. Petcu L. Picu A. Suceveanu A. P. Salmen T.. (2019). Microbiota signatures in type-2 diabetic patients with chronic kidney disease—a pilot study. J. Mind Med. Sci. 6, 130–136. doi: 10.22543/7674.61.p130136

[ref48] Gryp T. Vanholder R. Vaneechoutte M. Glorieux G. (2017). p-cresyl sulfate. Toxins 9:52. doi: 10.3390/toxins9020052 28146081 PMC5331431

[ref49] Gu J. L. Huang W. Zhang W. Q. Zhao T. T. Gao C. L. Gan W. J.. (2019). Sodium butyrate alleviates high-glucose-induced renal glomerular endothelial cells damage via inhibiting pyroptosis. Int. Immunopharmacol. 75:105832. doi: 10.1016/j.intimp.2019.105832, PMID: 31473434

[ref50] Gui D. D. Luo W. Yan B. J. Ren Z. Tang Z. H. Liu L. S.. (2021). Effects of gut microbiota on atherosclerosis through hydrogen sulfide. Eur. J. Pharmacol. 896:173916. doi: 10.1016/j.ejphar.2021.173916, PMID: 33529724

[ref51] Gupta N. Buffa J. A. Roberts A. B. Sangwan N. Skye S. M. Li L.. (2020). Targeted inhibition of gut microbial trimethylamine N-oxide production reduces renal tubulointerstitial fibrosis and functional impairment in a murine model of chronic kidney disease. Arterioscler. Thromb. 40, 1239–1255. doi: 10.1161/atvbaha.120.314139, PMID: 32212854 PMC7203662

[ref52] Hamzé R. Delangre E. Tolu S. Moreau M. Janel N. Bailbé D.. (2022). Type 2 diabetes mellitus and Alzheimer's disease: shared molecular mechanisms and potential common therapeutic targets. Int. J. Mol. Sci. 23:15287. doi: 10.3390/ijms232315287, PMID: 36499613 PMC9739879

[ref53] He X. Sun J. P. Liu C. Yu X. Y. Li H. X. Zhang W. J.. (2022). Compositional alterations of gut microbiota in patients with diabetic kidney disease and type 2 diabetes mellitus. Diabetes Metab. Syndr. Obesity 15, 755–765. doi: 10.2147/dmso.s347805, PMID: 35280499 PMC8911313

[ref54] Heilig C. W. Deb D. K. Abdul A. Riaz H. James L. R. Salameh J.. (2013). GLUT1 regulation of the pro-sclerotic mediators of diabetic nephropathy. Am. J. Nephrol. 38, 39–49. doi: 10.1159/000351989, PMID: 23817135

[ref55] Hobby G. P. Karaduta O. Dusio G. F. Singh M. Zybailov B. L. Arthur J. M. (2019). Chronic kidney disease and the gut microbiome. Am. J. Physiol-Renal 316, F1211–F1217. doi: 10.1152/ajprenal.00298.2018 PMC6620595 30864840

[ref56] Holscher H. D. (2017). Dietary fiber and prebiotics and the gastrointestinal microbiota. Gut Microbes 8, 172–184. doi: 10.1080/19490976.2017.1290756 28165863 PMC5390821

[ref57] Hsu C. N. Hou C. Y. Chang C. I. Tain Y. L. (2022). Resveratrol butyrate ester protects adenine-treated rats against hypertension and kidney disease by regulating the gut–kidney axis. Antioxidants 11:83. doi: 10.3390/antiox11010083 PMC8772985 35052587

[ref58] Hu D. M. Liu W. B. Yu W. L. Huang L. H. Ji C. L. Liu X. S.. (2023). Psyllium seed husk regulates the gut microbiota and improves mucosal barrier injury in the colon to attenuate renal injury in 5/6 nephrectomy rats. Ren. Fail. 45:2197076. doi: 10.1080/0886022x.2023.2197076, PMID: 37017261 PMC10078125

[ref59] Hu Z. B. Lu J. Chen P. P. Lu C. C. Zhang J. X. Li X. Q.. (2020). Dysbiosis of intestinal microbiota mediates tubulointerstitial injury in diabetic nephropathy via the disruption of cholesterol homeostasis. Theranostics 10, 2803–2816. doi: 10.7150/thno.40571, PMID: 32194836 PMC7052905

[ref60] Hu P. Zong Q. F. Zhao Y. H. Gu H. T. Liu Y. Y. Gu F.. (2022). Lactoferrin attenuates intestinal barrier dysfunction and inflammation by modulating the MAPK pathway and gut microbes in mice. J. Nutr. 152, 2451–2460. doi: 10.1093/jn/nxac200, PMID: 36774111

[ref61] Huang W. Guo H. L. Deng X. Zhu T. T. Xiong J. F. Xu Y. H.. (2017). Short-chain fatty acids inhibit oxidative stress and inflammation in mesangial cells induced by high glucose and lipopolysaccharide. Exp. Clin. Endocrinol. Diabetes 125, 98–105. doi: 10.1055/s-0042-121493, PMID: 28049222

[ref62] Huang W. Man Y. Gao C. L. Zhou L. P. Gu J. L. Xu H. W.. (2020). Short-chain fatty acids ameliorate diabetic nephropathy via GPR43-mediated inhibition of oxidative stress and NF-κB signaling. Oxidative Med. Cell. Longev. 2020:4074832. doi: 10.1155/2020/4074832, PMID: 32831998 PMC7422068

[ref63] Huang Q. H. Xu L. Q. Liu Y. H. Wu J. Z. Wu X. Lai X. P.. (2017). Polydatin protects rat liver against ethanol-induced injury: involvement of CYP2E1/ROS/Nrf2 and TLR4/NF-κB p65 pathway. Evid. Based Complement. Alternat. Med. 2017, 1–14. doi: 10.1155/2017/7953850, PMID: 29250126 PMC5698823

[ref64] Hussain S. Habib A. Najmi A. K. (2019). Limited knowledge of chronic kidney disease among type 2 diabetes mellitus patients in India. Int. J. Environ. Res. Public Health 16:1443. doi: 10.3390/ijerph16081443 31018581 PMC6518175

[ref65] Jansson Sigfrids F. Dahlström E. H. Forsblom C. Sandholm N. Harjutsalo V. Taskinen M. R.. (2021). Remnant cholesterol predicts progression of diabetic nephropathy and retinopathy in type 1 diabetes. J. Intern. Med. 290, 632–645. doi: 10.1111/joim.13298, PMID: 33964025

[ref66] Jaye K. Li C. G. Chang D. Bhuyan D. J. (2022). The role of key gut microbial metabolites in the development and treatment of cancer. Gut Microbes 14:2038865. doi: 10.1080/19490976.2022.2038865 35220885 PMC8890435

[ref67] Ji C. L. Deng Y. S. Yang A. C. Lu Z. Y. Chen Y. Liu X. S.. (2020). Rhubarb Enema improved colon mucosal barrier injury in 5/6 nephrectomy rats may associate with gut microbiota modification. Front. Pharmacol. 11:1092. doi: 10.3389/fphar.2020.01092, PMID: 32848732 PMC7403201

[ref68] Ji C. L. Li Y. Mo Y. A. Lu Z. Y. Lu F. H. Lin Q. Z.. (2021). Rhubarb Enema decreases circulating trimethylamine N-oxide level and improves renal fibrosis accompanied with gut microbiota change in chronic kidney disease rats. Front. Pharmacol. 12:780924. doi: 10.3389/fphar.2021.780924, PMID: 34966280 PMC8710758

[ref69] Ji C. L. Lu F. H. Wu Y. C. Lu Z. Y. Mo Y. A. Han L. J.. (2022). Rhubarb Enema increasing short-chain fatty acids that improves the intestinal barrier disruption in CKD may be related to the regulation of gut dysbiosis. Biomed. Res. Int. 2022, 1–15. doi: 10.1155/2022/1896781, PMID: 35097110 PMC8794667

[ref70] Jiang H. R. Cai M. M. Shen B. Y. Wang Q. Zhang T. C. Zhou X. (2022). Synbiotics and gut microbiota: new perspectives in the treatment of type 2 diabetes mellitus. Foods 11:2438. doi: 10.3390/foods11162438 36010438 PMC9407597

[ref71] Jiang H. Y. Zhang Y. Xu D. Y. Wang Q. (2021). Probiotics ameliorates glycemic control of patients with diabetic nephropathy: a randomized clinical study. J. Clin. Lab. Anal. 35:e23650. doi: 10.1002/jcla.23650 33666270 PMC8059722

[ref72] Jo J. Oh J. Park C. (2020). Microbial community analysis using high-throughput sequencing technology: a beginner's guide for microbiologists. J. Microbiol. 58, 176–192. doi: 10.1007/s12275-020-9525-5 32108314

[ref73] Johansen K. L. Chertow G. M. Foley R. N. Gilbertson D. T. Herzog C. A. Ishani A.. (2021). US renal data system 2020 annual data report: epidemiology of kidney disease in the United States. Am. J. Kidney Dis. 77, A7–A8. doi: 10.1053/j.ajkd.2021.01.002, PMID: 33752804 PMC8148988

[ref74] Jung C. Y. Yoo T. H. (2022). Pathophysiologic mechanisms and potential biomarkers in diabetic kidney disease. Diabetes Metab. J. 46, 181–197. doi: 10.4093/dmj.2021.0329 35385633 PMC8987689

[ref75] Kamada N. Chen G. Y. Inohara N. Nunez G. (2013). Control of pathogens and pathobionts by the gut microbiota. Nat. Immunol. 14, 685–690. doi: 10.1038/ni.2608 23778796 PMC4083503

[ref76] Khan I. Huang Z. B. Liang L. Y. Li N. Ali Z. Ding L.. (2021). Ammonia stress influences intestinal histomorphology, immune status and microbiota of Chinese striped-neck turtle (*Mauremys sinensis*). Ecotoxicol. Environ. Saf. 222:112471. doi: 10.1016/j.ecoenv.2021.112471, PMID: 34229168

[ref77] Kikuchi K. Saigusa D. Kanemitsu Y. Matsumoto Y. Thanai P. Suzuki N.. (2019). Gut microbiome-derived phenyl sulfate contributes to albuminuria in diabetic kidney disease. Nat. Commun. 10:1835. doi: 10.1038/s41467-019-09735-4, PMID: 31015435 PMC6478834

[ref78] Koppe L. Mafra D. Fouque D. (2015). Probiotics and chronic kidney disease. Kidney Int. 88, 958–966. doi: 10.1038/ki.2015.255 26376131

[ref79] Kundu S. Pushpakumar S. Sen U. (2015). MMP-9-and NMDA receptor-mediated mechanism of diabetic renovascular remodeling and kidney dysfunction: hydrogen sulfide is a key modulator. Nitric Oxide-Biol. Ch. 46, 172–185. doi: 10.1016/j.niox.2015.02.003 PMC4367483 25659756

[ref80] Lambert J. E. Myslicki J. P. Bomhof M. R. Belke D. D. Shearer J. Reimer R. A. (2015). Exercise training modifies gut microbiota in normal and diabetic mice. Appl. Physiol. Nutr. Me. 40, 749–752. doi: 10.1139/apnm-2014-0452 25962839

[ref81] Landis R. C. Quimby K. R. Greenidge A. R. (2018). M1/M2 macrophages in diabetic nephropathy: Nrf2/HO-1 as therapeutic targets. Curr. Pharm. Des. 24, 2241–2249. doi: 10.2174/1381612824666180716163845 30014796

[ref82] Lee J. W. Cowley E. S. Wolf P. G. Doden H. L. Murai T. Caicedo K. Y. O.. (2022). Formation of secondary Allo-bile acids by novel enzymes from gut Firmicutes. Gut Microbes 14:1. doi: 10.1080/19490976.2022.2132903, PMID: 36343662 PMC9645264

[ref83] Lee C. J. Sears C. L. Maruthur N. (2020). Gut microbiome and its role in obesity and insulin resistance. Ann. N. Y. Acad. Sci. 1461, 37–52. doi: 10.1111/nyas.14107 31087391

[ref84] Lei C. Sun R. Xu G. Tan Y. Feng W. McClain C. J.. (2022). Enteric VIP-producing neurons maintain gut microbiota homeostasis through regulating epithelium fucosylation. Cell Host Microbe 30, 1417–1434.e8. doi: 10.1016/j.chom.2022.09.001, PMID: 36150396 PMC9588764

[ref85] Li Y. J. Chen X. C. Kwan T. K. Loh Y. W. Singer J. Liu Y. Z.. (2020). Dietary fiber protects against diabetic nephropathy through short-chain fatty acid? Mediated activation of G protein? Coupled receptors GPR43 and GPR109A. J. Am. Soc. Nephrol. 31, 1267–1281. doi: 10.1681/asn.2019101029 32358041 PMC7269358

[ref86] Li S.-Y. Chen S. Lu X.-T. Fang A.-P. Chen Y.-M. Huang R.-Z.. (2022). Serum trimethylamine-N-oxide is associated with incident type 2 diabetes in middle-aged and older adults: a prospective cohort study. J. Transl. Med. 20:374. doi: 10.1186/s12967-022-03581-7, PMID: 35982495 PMC9389664

[ref87] Li H. Feng S. J. Zhang G. Z. Wang S. X. (2014). Correlation of lower concentrations of hydrogen sulfide with atherosclerosis in chronic hemodialysis patients with diabetic nephropathy. Blood Purif. 38, 188–194. doi: 10.1159/000368883 25531647

[ref88] Li Y. Qin G. Q. Wang W. Y. Liu X. Gao X. Q. Liu J. H.. (2022). Short chain fatty acids for the risk of diabetic nephropathy in type 2 diabetes patients. Acta Diabetol. 59, 901–909. doi: 10.1007/s00592-022-01870-7, PMID: 35368224

[ref89] Li Y. Su X. H. Gao Y. Lv C. X. Gao Z. W. Liu Y. P.. (2020). The potential role of the gut microbiota in modulating renal function in experimental diabetic nephropathy murine models established in same environment. BBA-Mol. Basis Dis. 1866:165764. doi: 10.1016/j.bbadis.2020.165764, PMID: 32169506

[ref90] Li L. Wei T. Liu S. Wang C. Zhao M. Feng Y.. (2021). Complement C5 activation promotes type 2 diabetic kidney disease via activating STAT3 pathway and disrupting the gut–kidney axis. J. Cell. Mol. Med. 25, 960–974. doi: 10.1111/jcmm.16157, PMID: 33280239 PMC7812276

[ref91] Lin M. Yiu W. H. Wu H. J. Chan L. Y. Y. Leung J. C. K. Au W. S.. (2012). Toll-like receptor 4 promotes tubular inflammation in diabetic nephropathy. J. Am. Soc. Nephrol. 23, 86–102. doi: 10.1681/asn.2010111210 22021706 PMC3269929

[ref92] Linh H. T. Iwata Y. Senda Y. Sakai-Takemori Y. Nakade Y. Oshima M.. (2022). Intestinal bacterial translocation contributes to diabetic kidney disease. J. Am. Soc. Nephrol. 33, 1105–1119. doi: 10.1681/asn.2021060843, PMID: 35264456 PMC9161796

[ref93] Liu J. Gao L. D. Fu B. Yang H. T. Zhang L. Che S. Q.. (2022). Efficacy and safety of Zicuiyin decoction on diabetic kidney disease: a multicenter, randomized controlled trial. Phytomedicine 100:154079. doi: 10.1016/j.phymed.2022.154079, PMID: 35413644

[ref94] Liu J. R. Miao H. Deng D. Q. Vaziri N. D. Li P. Zhao Y. Y. (2021). Gut microbiota-derived tryptophan metabolism mediates renal fibrosis by aryl hydrocarbon receptor signaling activation. Cell. Mol. Life Sci. 78, 909–922. doi: 10.1007/s00018-020-03645-1 32965514 PMC11073292

[ref95] Liu Y. X. Uruno A. Saito R. Matsukawa N. Hishinuma E. Saigusa D.. (2022). Nrf2 deficiency deteriorates diabetic kidney disease in Akita model mice. Redox Biol. 58:102525. doi: 10.1016/j.redox.2022.102525, PMID: 36335764 PMC9641024

[ref96] Liu L. Xia R. Song X. Q. Zhang B. P. He W. T. Zhou X. R.. (2021). Association between the triglyceride-glucose index and diabetic nephropathy in patients with type 2 diabetes: a cross-sectional study. J. Diabetes Investig. 12, 557–565. doi: 10.1111/jdi.13371, PMID: 33319507 PMC8015837

[ref97] Lobel L. Cao Y. G. Fenn K. Glickman J. N. Garrett W. S. (2020). Diet posttranslationally modifies the mouse gut microbial proteome to modulate renal function. Science 369, 1518–1531. doi: 10.1126/science.abb3763 32943527 PMC8178816

[ref98] Lodhi A. H. Ahmad F. U. D. Furwa K. Madni A. (2021). Role of oxidative stress and reduced endogenous hydrogen sulfide in diabetic nephropathy. Drug Des. Dev. Ther. 15, 1031–1043. doi: 10.2147/dddt.s291591 PMC7943325 33707940

[ref99] Lu J. Chen P. Zhang J. Li X. Wang G. Yuan B.. (2021). GPR43 deficiency protects against podocyte insulin resistance in diabetic nephropathy through the restoration of AMPKα activity. Theranostics 11, 4728–4742. doi: 10.7150/thno.56598, PMID: 33754024 PMC7978296

[ref100] Lu P. C. Hsu C. N. Lin I. C. Lo M. H. Yang M. Y. Tain Y. L. (2021). The association between changes in plasma short-chain fatty acid concentrations and hypertension in children with chronic kidney disease. Front. Pediatr. 8:613641. doi: 10.3389/fped.2020.613641 33614542 PMC7890123

[ref101] Lu C. C. Hu Z. B. Wang R. Hong Z. H. Lu J. Chen P. P.. (2020). Gut microbiota dysbiosis-induced activation of the intrarenal renin-angiotensin system is involved in kidney injuries in rat diabetic nephropathy. Acta Pharmacol. Sin. 41, 1111–1118. doi: 10.1038/s41401-019-0326-5, PMID: 32203081 PMC7471476

[ref102] Lu Y. D. Zhang Y. Zhao X. Shang C. Xiang M. Li L.. (2022). Microbiota-derived short-chain fatty acids: implications for cardiovascular and metabolic disease. Front. Cardiovasc. Med. 9:900381. doi: 10.3389/fcvm.2022.900381 36035928 PMC9403138

[ref103] Luo W. Guo S. Zhou Y. Zhu J. Zhao J. Wang M.. (2022). Hepatocellular carcinoma: novel understandings and therapeutic strategies based on bile acids (review). Int. J. Oncol. 61:117. doi: 10.3892/ijo.2022.5407, PMID: 35929515 PMC9450808

[ref104] Luo L. M. Luo J. L. Cai Y. T. Fu M. L. Li W. H. Shi L. L.. (2022). Inulin-type fructans change the gut microbiota and prevent the development of diabetic nephropathy. Pharmacol. Res. 183:106367. doi: 10.1016/j.phrs.2022.106367 35882293

[ref105] Lv Q. L. Li Z. Y. Sui A. H. Yang X. M. Han Y. F. Yao R. Y. (2022). The role and mechanisms of gut microbiota in diabetic nephropathy, diabetic retinopathy and cardiovascular diseases. Front. Microbiol. 13:977187. doi: 10.3389/fmicb.2022.977187 36060752 PMC9433831

[ref106] Mafi A. Namazi G. Soleimani A. Bahmani F. Aghadavod E. Asemi Z. (2018). Metabolic and genetic response to probiotics supplementation in patients with diabetic nephropathy: a randomized, double-blind, placebo-controlled trial. Food Funct. 9, 4763–4770. doi: 10.1039/c8fo00888d 30113051

[ref107] Makki K. Brolin H. Petersen N. Henricsson M. Christensen D. P. Khan M. T.. (2023). 6α-hydroxylated bile acids mediate TGR5 signalling to improve glucose metabolism upon dietary fiber supplementation in mice. Gut 72, 314–324. doi: 10.1136/gutjnl-2021-326541, PMID: 35697422 PMC9872241

[ref108] Malek V. Suryavanshi S. V. Sharma N. Kulkarni Y. A. Mulay S. R. Gaikwad A. B. (2021). Potential of renin-angiotensin-aldosterone system modulations in diabetic kidney disease: old players to new hope! Rev. Physiol. Bioch. P. 179, 31–71. doi: 10.1007/112_2020_50 32979084

[ref109] Mansoor G. Tahir M. Maqbool T. Abbasi S. Q. Hadi F. Shakoori T. A.. (2022). Increased expression of circulating stress markers, inflammatory cytokines and decreased antioxidant level in diabetic nephropathy. Lietuvis̆koji medicina 58:1604. doi: 10.3390/medicina58111604, PMID: 36363561 PMC9694611

[ref110] Masaoutis C. Theocharis S. (2019). The farnesoid X receptor: a potential target for expanding the therapeutic arsenal against kidney disease. Expert Opin. Ther. Targets 23, 107–116. doi: 10.1080/14728222.2019.1559825 30577722

[ref111] Meijers B. K. I. Evenepoel P. (2011). The gut–kidney axis: indoxyl sulfate, p-cresyl sulfate and CKD progression. Nephrol. Dial. Transpl. 26, 759–761. doi: 10.1093/ndt/gfq818 21343587

[ref112] Mezzano S. Aros C. Droguett A. Burgos M. E. Ardiles L. Flores C.. (2004). NF- B activation and overexpression of regulated genes in human diabetic nephropathy. Nephrol. Dial. Transplant. 19, 2505–2512. doi: 10.1093/ndt/gfh207, PMID: 15280531

[ref113] Miraghajani M. Zaghian N. Dehkohneh A. Mirlohi M. Ghiasvand R. (2019). Probiotic soy milk consumption and renal function among type 2 diabetic patients with nephropathy: a randomized controlled clinical trial. Probiotics Antimicro. 11, 124–132. doi: 10.1007/s12602-017-9325-3 28884306

[ref114] Miraghajani M. Zaghian N. Mirlohi M. Feizi A. Ghiasvand R. (2017). The impact of probiotic soy milk consumption on oxidative stress among type 2 diabetic kidney disease patients: a randomized controlled clinical trial. J. Ren. Nutr. 27, 317–324. doi: 10.1053/j.jrn.2017.04.004 28579313

[ref115] Moniri N. H. Farah Q. (2021). Short-chain free-fatty acid G protein-coupled receptors in colon cancer. Biochem. Pharmacol. 186:114483. doi: 10.1016/j.bcp.2021.114483 33631190

[ref116] Mutsaers H. A. M. Caetano-Pinto P. Seegers A. E. M. Dankers A. C. A. van den Broek P. H. H. Wetzels J. F. M.. (2015). Proximal tubular efflux transporters involved in renal excretion of p-cresyl sulfate and p-cresyl glucuronide: implications for chronic kidney disease pathophysiology. Toxicol. In Vitro 29, 1868–1877. doi: 10.1016/j.tiv.2015.07.020, PMID: 26216510

[ref117] Naimi S. Viennois E. Gewirtz A. T. Chassaing B. (2021). Direct impact of commonly used dietary emulsifiers on human gut microbiota. Microbiome 9:66. doi: 10.1186/s40168-020-00996-6 33752754 PMC7986288

[ref118] Navarro-Gonzalez J. F. Mora-Fernandez C. de Fuentes M. M. Garcia-Perez J. (2011). Inflammatory molecules and pathways in the pathogenesis of diabetic nephropathy. Nat. Rev. Nephrol. 7, 327–340. doi: 10.1038/nrneph.2011.51 21537349

[ref119] Ni Y. Zheng L. Nan S. Ke L. Fu Z. Jin J. (2022). Enterorenal crosstalks in diabetic nephropathy and novel therapeutics targeting the gut microbiota. Acta bioch. bioph. Sin. 54, 1406–1420. doi: 10.3724/abbs.2022140 PMC9827797 36239349

[ref120] Niewczas M. A. Sirich T. L. Mathew A. V. Skupien J. Mohney R. P. Warram J. H.. (2014). Uremic solutes and risk of end-stage renal disease in type 2 diabetes: metabolomic study. Kidney Int. 85, 1214–1224. doi: 10.1038/ki.2013.497, PMID: 24429397 PMC4072128

[ref121] Niewiem M. Grzybowska-Chlebowczyk U. (2022). Intestinal barrier permeability in allergic diseases. Nutrients 14:1893. doi: 10.3390/nu14091893 35565858 PMC9101724

[ref122] Nishiyama A. Kobori H. (2018). Independent regulation of renin-angiotensin-aldosterone system in the kidney. Clin. Exp. Nephrol. 22, 1231–1239. doi: 10.1007/s10157-018-1567-1 29600408 PMC6163102

[ref123] Pasini E. Corsetti G. Assanelli D. Testa C. Romano C. Dioguardi F. S.. (2019). Effects of chronic exercise on gut microbiota and intestinal barrier in human with type 2 diabetes. Minerva Med. 110, 3–11. doi: 10.23736/s0026-4806.18.05589-1, PMID: 30667205

[ref124] Pavlov T. S. Palygin O. Isaeva E. Levchenko V. Khedr S. Blass G.. (2020). NOX4-dependent regulation of ENaC in hypertension and diabetic kidney disease. FASEB J. 34, 13396–13408. doi: 10.1096/fj.202000966RR, PMID: 32799394 PMC7722042

[ref125] Pengrattanachot N. Thongnak L. Lungkaphin A. (2022). The impact of prebiotic fructooligosaccharides on gut dysbiosis and inflammation in obesity and diabetes related kidney disease. Food Funct. 13, 5925–5945. doi: 10.1039/d1fo04428a 35583860

[ref126] Perino A. Demagny H. Velazquez-Villegas L. Schoonjans K. (2021). Molecular physiology of bile acid signaling in health, disease, and aging. Physiol. Rev. 101, 683–731. doi: 10.1152/physrev.00049.2019 32790577

[ref127] Pujari R. Banerjee G. (2021). Impact of prebiotics on immune response: from the bench to the clinic. Immunol. Cell Biol. 99, 255–273. doi: 10.1111/imcb.12409 32996638

[ref128] Qu Z. H. Tian P. J. Yang B. Zhao J. X. Wang G. Chen W. (2022). Fecal microbiota transplantation for diseases: therapeutic potential, methodology, risk management in clinical practice. Life Sci. 304:120719. doi: 10.1016/j.lfs.2022.120719 35716734

[ref129] Romano K. A. Vivas E. I. Amador-Noguez D. Rey F. E. (2015). Intestinal microbiota composition modulates choline bioavailability from diet and accumulation of the proatherogenic metabolite trimethylamine-N-oxide. MBio 6, e02481–e02414. doi: 10.1128/mBio.02481-14 25784704 PMC4453578

[ref130] Roscioni S. S. Heerspink H. J. L. de Zeeuw D. (2014). The effect of RAAS blockade on the progression of diabetic nephropathy. Nat. Rev. Nephrol. 10, 77–87. doi: 10.1038/nrneph.2013.251 24296623

[ref131] Rossi M. Johnson D. W. Morrison M. Pascoe E. M. Coombes J. S. Forbes J. M.. (2016). Synbiotics easing renal failure by improving gut microbiology (SYNERGY): a randomized trial. Clin. J. Am. Soc. Nephrol. 11, 223–231. doi: 10.2215/cjn.05240515, PMID: 26772193 PMC4741035

[ref132] Rustiasari U. J. Roelofs J. J. (2022). The role of platelets in diabetic kidney disease. Int. J. Mol. Sci. 23:8270. doi: 10.3390/ijms23158270 35955405 PMC9368651

[ref133] Salguero M. V. Al-Obaide M. A. I. Singh R. Siepmann T. Vasylyeva T. L. (2019). Dysbiosis of gram-negative gut microbiota and the associated serum lipopolysaccharide exacerbates inflammation in type 2 diabetic patients with chronic kidney disease. Exp. Ther. Med. 18, 3461–3469. doi: 10.3892/etm.2019.7943 31602221 PMC6777309

[ref134] Samsu N. (2021). Diabetic nephropathy: challenges in pathogenesis, diagnosis, and treatment. Biomed. Res. Int. 2021:1497449. doi: 10.1155/2021/1497449 34307650 PMC8285185

[ref135] Shen H. J. Wang W. (2021). Effect of glutathione liposomes on diabetic nephropathy based on oxidative stress and polyol pathway mechanism. J. Liposome Res. 31, 317–325. doi: 10.1080/08982104.2020.1780607 32567425

[ref136] Sheng Y. Zheng S. J. Zhang C. H. Zhao C. H. He X. Y. Xu W. T.. (2018). Mulberry leaf tea alleviates diabetic nephropathy by inhibiting PKC signaling and modulating intestinal flora. J. Funct. Foods 46, 118–127. doi: 10.1016/j.jff.2018.04.040

[ref137] Shi R. Y. Tao Y. J. Tang H. T. Wu C. H. Fei J. J. Ge H. T.. (2023). *Abelmoschus Manihot* ameliorates the levels of circulating metabolites in diabetic nephropathy by modulating gut microbiota in non-obese diabetes mice. Microb. Biotechnol. 16, 813–826. doi: 10.1111/1751-7915.14200, PMID: 36583468 PMC10034626

[ref138] Silva A. Lanza K. Palmeira V. A. Costa L. B. Flynn J. T. (2021). 2020 update on the renin-angiotensin-aldosterone system in pediatric kidney disease and its interactions with coronavirus. Pediatr. Nephrol. 36, 1407–1426. doi: 10.1007/s00467-020-04759-1 32995920 PMC7524035

[ref139] Silwal P. Kim I. S. Jo E. K. (2021). Autophagy and host defense in nontuberculous mycobacterial infection. Front. Immunol. 12:728742. doi: 10.3389/fimmu.2021.728742 34552591 PMC8450401

[ref140] Singh R. P. Shadan A. Ma Y. (2022). Biotechnological applications of probiotics: a multifarious weapon to disease and metabolic abnormality. Probiotics Antimicro. 14, 1184–1210. doi: 10.1007/s12602-022-09992-8 PMC9483357 36121610

[ref141] Smith E. A. Macfarlane G. T. (1996). Enumeration of human colonic bacteria producing phenolic and indolic compounds: effects of pH, carbohydrate availability and retention time on dissimilatory aromatic amino acid metabolism. J. Appl. Bacteriol. 81, 288–302. doi: 10.1111/j.1365-2672.1996.tb04331.x 8810056

[ref142] Spindler M. P. Siu S. Mogno I. Li Z. Yang C. Mehandru S.. (2022). Human gut microbiota stimulate defined innate immune responses that vary from phylum to strain. Cell Host Microbe 30, 1481–1498.e5. doi: 10.1016/j.chom.2022.08.009, PMID: 36099923 PMC9588646

[ref143] Stavropoulou E. Kantartzi K. Tsigalou C. Konstantinidis T. Romanidou G. Voidarou C.. (2021). Focus on the gut–kidney axis in health and disease. Front. Med. 7:620102. doi: 10.3389/fmed.2020.620102, PMID: 33553216 PMC7859267

[ref144] Su S. Ma Z. Wu H. Xu Z. Yi H. (2023). Oxidative stress as a culprit in diabetic kidney disease. Life Sci. 322:121661. doi: 10.1016/j.lfs.2023.121661 37028547

[ref145] Su X. H. Yu W. X. Liu A. R. Wang C. X. Li X. Z. Gao J. J.. (2022). San-Huang-Yi-Shen capsule ameliorates diabetic nephropathy in rats through modulating the gut microbiota and overall metabolism. Front. Pharmacol. 12:808867. doi: 10.3389/fphar.2021.808867 35058786 PMC8764181

[ref146] Sun X. Chen J. Huang Y. T. Zhu S. Wang S. S. Xu Z. J.. (2022). Yishen Qingli Heluo granule ameliorates renal dysfunction in 5/6 nephrectomized rats by targeting gut microbiota and intestinal barrier integrity. Front. Pharmacol. 13:858881. doi: 10.3389/fphar.2022.858881 35814258 PMC9258868

[ref147] Sun L. Kanwar Y. S. (2015). Relevance of TNF-alpha in the context of other inflammatory cytokines in the progression of diabetic nephropathy. Kidney Int. 88, 662–665. doi: 10.1038/ki.2015.250 26422621 PMC4589869

[ref148] Sun R. Y. Mu H. H. Sun M. B. Miao X. Y. Xin J. Xu X. F.. (2022). Effects of *Bacillus subtilis* natto JLCC513 on gut microbiota and intestinal barrier function in obese rats. J. Appl. Microbiol. 133, 3634–3644. doi: 10.1111/jam.15797 36036228

[ref149] Sun H. Saeedi P. Karuranga S. Pinkepank M. Ogurtsova K. Duncan B. B.. (2022). IDF diabetes atlas: global, regional and country-level diabetes prevalence estimates for 2021 and projections for 2045. Diabetes Res. Clin. Pract. 183:109119. doi: 10.1016/j.diabres.2021.109119, PMID: 34879977 PMC11057359

[ref150] Tan Y. Q. Wang Y. N. Feng H. Y. Guo Z. Y. Li X. Nie X. L.. (2022). Host/microbiota interactions-derived tryptophan metabolites modulate oxidative stress and inflammation via aryl hydrocarbon receptor signaling. Free Radic. Biol. Med. 184, 30–41. doi: 10.1016/j.freeradbiomed.2022.03.025, PMID: 35367341

[ref151] Tanase D. M. Gosav E. M. Anton M. I. Floria M. Seritean Isac P. N. Hurjui L. L.. (2022). Oxidative stress and NRF2/KEAP1/ARE pathway in diabetic kidney disease (DKD): new perspectives. Biomol. Ther. 12:1227. doi: 10.3390/biom12091227, PMID: 36139066 PMC9496369

[ref152] Tao S. B. Li L. Z. Li L. Liu Y. Ren Q. Shi M.. (2019). Understanding the gut–kidney axis among biopsy-proven diabetic nephropathy, type 2 diabetes mellitus and healthy controls: an analysis of the gut microbiota composition. Acta Diabetol. 56, 581–592. doi: 10.1007/s00592-019-01316-7, PMID: 30888537

[ref153] Thibaut M. M. Bindels L. B. (2022). Crosstalk between bile acid-activated receptors and microbiome in entero-hepatic inflammation. Trends Mol. Med. 28, 223–236. doi: 10.1016/j.molmed.2021.12.006 35074252

[ref154] Tuttle K. R. Agarwal R. Alpers C. E. Bakris G. L. Brosius F. C. Kolkhof P.. (2022). Molecular mechanisms and therapeutic targets for diabetic kidney disease. Kidney Int. 102, 248–260. doi: 10.1016/j.kint.2022.05.012 35661785

[ref155] van der Hee B. Wells J. M. (2021). Microbial regulation of host physiology by short-chain fatty acids. Trends Microbiol. 29, 700–712. doi: 10.1016/j.tim.2021.02.001 33674141

[ref156] Vaziri N. D. Yuan J. Norris K. (2013). Role of urea in intestinal barrier dysfunction and disruption of epithelial tight junction in chronic kidney disease. Am. J. Nephrol. 37, 1–6. doi: 10.1159/000345969 23258127 PMC3686571

[ref157] Waller K. M. J. Leong R. W. Paramsothy S. (2022). An update on fecal microbiota transplantation for the treatment of gastrointestinal diseases. J. Gastroenterol. Hepatol. 37, 246–255. doi: 10.1111/jgh.15731 34735024

[ref158] Wang M. Chen F. Q. Wang J. L. Zeng Z. X. Yang Q. Shao S. Y. (2018). Th17 and Treg lymphocytes in obesity and type 2 diabetic patients. Clin. Immunol. 197, 77–85. doi: 10.1016/j.clim.2018.09.005 30218707

[ref159] Wang X. X. X. Edelstein M. H. Gafter U. Qiu L. R. Luo Y. H. Dobrinskikh E.. (2016). G protein-coupled bile acid receptor TGR5 activation inhibits kidney disease in obesity and diabetes. J. Am. Soc. Nephrol. 27, 1362–1378. doi: 10.1681/asn.2014121271, PMID: 26424786 PMC4849814

[ref160] Wang X. X. X. Jiang T. Shen Y. Caldas Y. Miyazaki-Anzai S. Santamaria H.. (2010). Diabetic nephropathy is accelerated by farnesoid X receptor deficiency and inhibited by farnesoid X receptor activation in a type 1 diabetes model. Diabetes 59, 2916–2927. doi: 10.2337/db10-0019, PMID: 20699418 PMC2963551

[ref161] Wang H. Lu Y. Yan Y. Tian S. S. Zheng D. J. Leng D. J.. (2020). Promising treatment for type 2 diabetes: fecal microbiota transplantation reverses insulin resistance and impaired islets. FCIMB 9:455. doi: 10.3389/fcimb.2019.00455, PMID: 32010641 PMC6979041

[ref162] Wang L. Mu X. Chen X. Han Y. (2022). Hydrogen sulfide attenuates intracellular oxidative stress via repressing glycolate oxidase activities in *Arabidopsis thaliana*. BMC Plant Biol. 22:98. doi: 10.1186/s12870-022-03490-3 35247968 PMC8897949

[ref163] Wang H. Q. Wang S. S. Kuok C. Wang Q. Cheng X. L. (2019). Umbelliferone ameliorates renal function in diabetic nephropathy rats through regulating inflammation and TLR/NF-κ B pathway. Chin. J. Nat. Medicines 17, 346–354. doi: 10.1016/s1875-5364(19)30040-8 31171269

[ref164] Wang X. X. X. Wang D. Luo Y. H. Myakala K. Dobrinskikh E. Rosenberg A. Z.. (2018). FXR/TGR5 dual agonist prevents progression of nephropathy in diabetes and obesity. J. Am. Soc. Nephrol. 29, 118–137. doi: 10.1681/asn.2017020222, PMID: 29089371 PMC5748904

[ref165] Wang J. M. Xiang H. J. Lu Y. F. Wu T. Ji G. (2021). New progress in drugs treatment of diabetic kidney disease. Biomed. Pharmacother. 141:111918. doi: 10.1016/j.biopha.2021.111918 34328095

[ref166] Wang S. P. Zhang W. Cao A. Z. Pan Z. C. Liu T. L. (2022). Dietary supplementation of bile acids in tongue sole (*Cynoglossus semilaevis*): a promising strategy to improve hepatic health status. Front. Mar. Sci. 9:899768. doi: 10.3389/fmars.2022.899768

[ref167] Wang Y. W. Zhao J. Qin Y. L. Yu Z. X. Zhang Y. M. Ning X. X.. (2022). The specific alteration of gut microbiota in diabetic kidney diseases-a systematic review and meta-analysis. Front. Immunol. 13:908219. doi: 10.3389/fimmu.2022.908219, PMID: 35784273 PMC9248803

[ref168] Wang F. Z. Zhou H. Wang H. Y. Dai H. B. Gao Q. Qian P.. (2021). Hydrogen sulfide prevents arterial medial calcification in rats with diabetic nephropathy. BMC Cardiovasc. Disord. 21:495. doi: 10.1186/s12872-021-02307-9 34645391 PMC8515673

[ref169] Warren A. M. Knudsen S. T. Cooper M. E. (2019). Diabetic nephropathy: an insight into molecular mechanisms and emerging therapies. Expert Opin. Ther. Targets 23, 579–591. doi: 10.1080/14728222.2019.1624721 31154867

[ref170] Wegierska A. E. Charitos I. A. Topi S. Potenza M. A. Montagnani M. Santacroce L. (2022). The connection between physical exercise and gut microbiota: implications for competitive sports athletes. Sports Med. 52, 2355–2369. doi: 10.1007/s40279-022-01696-x 35596883 PMC9474385

[ref171] Wei H. Wang L. An Z. Xie H. Liu W. du Q.. (2021). QiDiTangShen granules modulated the gut microbiome composition and improved bile acid profi les in a mouse model of diabetic nephropathy. Biomed. Pharmacother. 133:111061. doi: 10.1016/j.biopha.2020.111061, PMID: 33378964

[ref172] Wei C. W. Wu T. K. Wu S. C. Chen Y. L. Pan Y. R. Chien Y. C.. (2022). Curcumin enhances p-cresyl sulfate-induced cytotoxic effects on renal tubular cells. Int. J. Med. Sci. 19, 1138–1146. doi: 10.7150/ijms.72646, PMID: 35919818 PMC9339410

[ref173] Williams B. M. Cliff C. L. Lee K. Squires P. E. Hills C. E. (2022). The role of the NLRP3 Inflammasome in mediating glomerular and tubular injury in diabetic nephropathy. Front. Physiol. 13:907504. doi: 10.3389/fphys.2022.907504 35755447 PMC9218738

[ref174] Winiarska A. Knysak M. Nabrdalik K. Gumprecht J. Stompor T. (2021). Inflammation and oxidative stress in diabetic kidney disease: the targets for SGLT2 inhibitors and GLP-1 receptor agonists. Int. J. Mol. Sci. 22:10822. doi: 10.3390/ijms221910822 34639160 PMC8509708

[ref175] Wu C. Fei J. Xu Q. Tao Y. Zhou Z. Wang Y.. (2022). Interaction between plasma metabolomics and intestinal microbiome in db/db mouse, an animal model for study of type 2 diabetes and diabetic kidney disease. Meta 12:775. doi: 10.3390/metabo12090775, PMID: 36144180 PMC9503368

[ref176] Wu T. Y. Wang G. Q. Xiong Z. Q. Xia Y. J. Song X. Zhang H.. (2022). Probiotics interact with lipids metabolism and affect gut health. Front. Nutr. 9:917043. doi: 10.3389/fnut.2022.917043, PMID: 35711544 PMC9195177

[ref177] Wu X. Q. Zhang D. D. Wang Y. N. Tan Y. Q. Yu X. Y. Zhao Y. Y. (2021). AGE/RAGE in diabetic kidney disease and ageing kidney. Free Radic. Biol. Med. 171, 260–271. doi: 10.1016/j.freeradbiomed.2021.05.025 34019934

[ref178] Xiao H. M. Sun X. H. Liu R. B. Chen Z. Q. Lin Z. Y. Yang Y.. (2020). Gentiopicroside activates the bile acid receptor Gpbar1 (TGR5) to repress NF-κ B pathway and ameliorate diabetic nephropathy. Pharmacol. Res. 151:104559. doi: 10.1016/j.phrs.2019.104559 31759089

[ref179] Xie Y. Lin X. Yuan J. Dong R. Yu J. L. Zha Y. (2022). Effects of acteoside on the expressions of MCP-1 and TGF-β (1) in the diabetic nephropathy mice. Eur. J. Inflamm. 20:1721727X221118348. doi: 10.1177/1721727x221118348

[ref180] Xiong F. X. Li X. J. Yang Z. Y. Wang Y. Gong W. Y. Huang J. Y.. (2016). TGR5 suppresses high glucose-induced upregulation of fibronectin and transforming growth factor-β 1 in rat glomerular mesangial cells by inhibiting RhoA/ROCK signaling. Endocrine 54, 657–670. doi: 10.1007/s12020-016-1032-4 27470217

[ref181] Xu Y. H. Gao C. L. Guo H. L. Zhang W. Q. Huang W. Tang S. S.. (2018). Sodium butyrate supplementation ameliorates diabetic inflammation in db/db mice. J. Endocrinol. 238, 231–244. doi: 10.1530/joe-18-0137, PMID: 29941502

[ref182] Xu J. Yao X. Z. Li X. Y. Xie S. W. Chi S. Y. Zhang S.. (2022). Bile acids reduced the lipid deposition in fatty degenerated hepatocytes of pearl gentian grouper (*Epinephelus fuscoguttatus*♂ × *Epinephelus lanceolatus*♀) in vitro. Front. Mar. Sci. 9:861117. doi: 10.3389/fmars.2022.861117

[ref183] Yamamoto J. Sato W. Kosugi T. Yamamoto T. Kimura T. Taniguchi S.. (2013). Distribution of hydrogen sulfide (H_2_S)-producing enzymes and the roles of the H2S donor sodium hydrosulfide in diabetic nephropathy. Clin. Exp. Nephrol. 17, 32–40. doi: 10.1007/s10157-012-0670-y, PMID: 22872231 PMC3572382

[ref184] Yang C. Y. Chen T. W. Lu W. L. Liang S. S. Huang H. D. Tseng C. P.. (2021). Synbiotics alleviate the gut indole load and dysbiosis in chronic kidney disease. Cells 10:114. doi: 10.3390/cells10010114, PMID: 33435396 PMC7826693

[ref185] Yang J. Dong H. Wang Y. Jiang Y. Zhang W. Lu Y.. (2020). Cordyceps cicadae polysaccharides ameliorated renal interstitial fibrosis in diabetic nephropathy rats by repressing inflammation and modulating gut microbiota dysbiosis. Int. J. Biol. Macromol. 163, 442–456. doi: 10.1016/j.ijbiomac.2020.06.153, PMID: 32592781

[ref186] Yang L. Lin H. Q. Lin W. T. Xu X. Y. (2020). Exercise ameliorates insulin resistance of type 2 diabetes through motivating short-chain fatty acid-mediated skeletal muscle cell autophagy. Biology-Basel 9:9080203. doi: 10.3390/biology9080203 PMC7464264 32756447

[ref187] Yang Z. Y. Xiong F. X. Wang Y. Gong W. Y. Huang J. Y. Chen C.. (2016). TGR5 activation suppressed S1P/S1P2 signaling and resisted high glucose-induced fibrosis in glomerular mesangial cells. Pharmacol. Res. 111, 226–236. doi: 10.1016/j.phrs.2016.05.035, PMID: 27317945

[ref188] Yang Y. Xu G. S. (2022). Update on pathogenesis of glomerular hyperfiltration in early diabetic kidney disease. Front. Endocrinol. 13:872918. doi: 10.3389/fendo.2022.872918 PMC9161673 35663316

[ref189] Yang H. Xu W. Zhou Z. Liu J. Li X. Chen L.. (2015). Curcumin attenuates urinary excretion of albumin in type II diabetic patients with enhancing nuclear factor erythroid-derived 2-like 2 (Nrf2) system and repressing inflammatory signaling efficacies. Exp. Clin. Endocrinol. Diabetes 123, 360–367. doi: 10.1055/s-0035-1545345, PMID: 25875220

[ref190] Ying L. W. Shen Y. Zhang Y. Wang Y. K. Liu Y. Yin J.. (2021). Association of advanced glycation end products with diabetic retinopathy in type 2 diabetes mellitus. Diabetes Res. Clin. Pract. 177:108880. doi: 10.1016/j.diabres.2021.108880 34058298

[ref191] Yoo W. Zieba J. Foegeding N. Torres T. Shelton C. Shealy N.. (2021). High-fat diet–induced colonocyte dysfunction escalates microbiota-derived trimethylamineN-oxide. Science 373, 813–818. doi: 10.1126/science.aba3683, PMID: 34385401 PMC8506909

[ref192] Yu N. Gu N. Wang Y. X. Zhou B. Lu D. F. Li J. P.. (2022). The association of plasma trimethylamine N-oxide with coronary atherosclerotic burden in patients with type 2 diabetes among a Chinese north population. Diabetes Metab. Syndr. Obesity 15, 69–78. doi: 10.2147/dmso.s339698, PMID: 35035225 PMC8754460

[ref193] Zafar H. Saier M. H. (2021). Gut Bacteroides species in health and disease. Gut Microbes 13:1848158. doi: 10.1080/19490976.2020.1848158 33535896 PMC7872030

[ref194] Zakrzewska Z. Zawartka A. Schab M. Martyniak A. Skoczeń S. Tomasik P. J.. (2022). Prebiotics, probiotics, and postbiotics in the prevention and treatment of anemia. Microorganisms 10:1330. doi: 10.3390/microorganisms10071330, PMID: 35889049 PMC9317605

[ref195] Zeng O. Li F. Li Y. Li L. Xiao T. Chu C.. (2016). Effect of novel gasotransmitter hydrogen sulfide on renal fibrosis and connexins expression in diabetic rats. Bioengineered 7, 314–320. doi: 10.1080/21655979.2016.1197743, PMID: 27575818 PMC5060972

[ref196] Zhang Z. P. Li Q. Y. Liu F. Li D. Y. (2022). Lycoperoside H protects against diabetic nephropathy via alteration of gut microbiota and inflammation. J. Biochem. Mol. Toxicol. 36:e23216. doi: 10.1002/jbt.23216 36156833

[ref197] Zhang F. Qi L. L. Feng Q. Y. Zhang B. K. Li X. Y. Liu C.. (2021). HIPK2 phosphorylates HDAC3 for NF-κ B acetylation to ameliorate colitis-associated colorectal carcinoma and sepsis. Proc. Natl. Acad. Sci. U. S. A. 118:e2021798118. doi: 10.1073/pnas.2021798118 34244427 PMC8285910

[ref198] Zhang B. Wan Y. Z. Zhou X. F. Zhang H. J. Zhao H. L. Ma L.. (2022). Characteristics of serum metabolites and gut microbiota in diabetic kidney disease. Front. Pharmacol. 13:872988. doi: 10.3389/fphar.2022.872988, PMID: 35548353 PMC9084235

[ref199] Zhang L. L. Wang Z. S. Zhang X. N. Zhao L. Chu J. J. Li H. B.. (2022). Alterations of the gut microbiota in patients with diabetic nephropathy. Microbiol. Spectr., e00324–e00322. doi: 10.1128/spectrum.00324-22 35863004 PMC9430528

[ref200] Zhang M. Yang L. C. Zhu M. M. Yang B. Yang Y. J. Jia X. B.. (2022). Moutan cortex polysaccharide ameliorates diabetic kidney disease via modulating gut microbiota dynamically in rats. Int. J. Biol. Macromol. 206, 849–860. doi: 10.1016/j.ijbiomac.2022.03.077 35307460

[ref201] Zhang Q. Zhang Y. Zeng L. Chen G. Zhang L. Liu M.. (2021). The role of gut microbiota and microbiota-related serum metabolites in the progression of diabetic kidney disease. Front. Pharmacol. 12:757508. doi: 10.3389/fphar.2021.757508, PMID: 34899312 PMC8652004

[ref202] Zhang L. Zhao M. H. Zuo L. (2020). China kidney disease network (CK-NET) 2015 annual data report. Kidney Int. Suppl. 10:E95. doi: 10.1016/j.kisu.2019.05.001 PMC6382959 30828481

[ref203] Zhao H. J. Liu H. Y. Yang Y. H. Lan T. Y. Wang H. G. Wu D. D. (2022). Hydrogen sulfide plays an important role by regulating endoplasmic reticulum stress in diabetes-related diseases. Int. J. Mol. Sci. 23:7170. doi: 10.3390/ijms23137170 35806174 PMC9266787

[ref204] Zhao J. Ning X. X. Liu B. J. Dong R. J. Bai M. Sun S. R. (2021). Specific alterations in gut microbiota in patients with chronic kidney disease: an updated systematic review. Ren. Fail. 43, 102–112. doi: 10.1080/0886022x.2020.1864404 33406960 PMC7808321

[ref205] Zhao J. Zhang Q. L. Shen J. H. Wang K. Liu J. (2019). Magnesium lithospermate B improves the gut microbiome and bile acid metabolic profiles in a mouse model of diabetic nephropathy. Acta Pharmacol. Sin. 40, 507–513. doi: 10.1038/s41401-018-0029-3 29941869 PMC6461985

[ref206] Zhao T. Zhang H. Yin X. Zhao H. Ma L. Yan M.. (2020). Tangshen formula modulates gut microbiota and reduces gut-derived toxins in diabetic nephropathy rats. Biomed. Pharmacother. 129:110325. doi: 10.1016/j.biopha.2020.110325, PMID: 32535383

[ref207] Zheng S. Y. Li H. X. Xu R. C. Miao W. T. Dai M. Y. Ding S. T.. (2021). Potential roles of gut microbiota and microbial metabolites in Parkinson's disease. Ageing Res. Rev. 69:101347. doi: 10.1016/j.arr.2021.101347, PMID: 33905953

[ref208] Zhong C. Y. Dai Z. W. Chai L. X. Wu L. P. Li J. H. Guo W. Y.. (2021). The change of gut microbiota-derived short-chain fatty acids in diabetic kidney disease. J. Clin. Lab. Anal. 35:e24062. doi: 10.1002/jcla.24062, PMID: 34689373 PMC8649351

[ref209] Zhong Y. F. Lee K. He J. C. (2018). SIRT1 is a potential drug target for treatment of diabetic kidney disease. Front. Endocrinol. 9:624. doi: 10.3389/fendo.2018.00624 PMC6199382 30386303

[ref210] Zhou B. S. Feng B. Qin Z. X. Zhao Y. G. Chen Y. Shi Z. M.. (2016). Activation of farnesoid X receptor downregulates visfatin and attenuates diabetic nephropathy. Mol. Cell. Endocrinol. 419, 72–82. doi: 10.1016/j.mce.2015.10.001, PMID: 26450152

[ref211] Zhou X. Feng Y. Zhan Z. B. Chen J. C. (2014). Hydrogen sulfide alleviates diabetic nephropathy in a streptozotocin-induced diabetic rat model. J. Biol. Chem. 289, 28827–28834. doi: 10.1074/jbc.M114.596593 25164822 PMC4200243

[ref212] Zhou T. T. Xu H. W. Cheng X. He Y. Q. Ren Q. Li D. Z.. (2022). Sodium butyrate attenuates diabetic kidney disease partially via histone butyrylation modification. Mediat. Inflamm. 2022:7643322. doi: 10.1155/2022/7643322, PMID: 35909658 PMC9329006

[ref213] Zhu W. F. Gregory J. C. Org E. Buffa J. A. Gupta N. Wang Z. N.. (2016). Gut microbial metabolite TMAO enhances platelet hyperreactivity and thrombosis risk. Cells 165, 111–124. doi: 10.1016/j.cell.2016.02.011 PMC4862743 26972052

[ref214] Zixin Y. Lulu C. Xiangchang Z. Qing F. Binjie Z. Chunyang L.. (2022). TMAO as a potential biomarker and therapeutic target for chronic kidney disease: a review. Front. Pharmacol. 13:929262. doi: 10.3389/fphar.2022.929262, PMID: 36034781 PMC9411716

